# Enhancement of Endocannabinoid Signaling by Dual MAGL/ABHD6 Inhibition Reduces Neurodegeneration and Neuro-Inflammation Following AMPA-Mediated Excitotoxicity

**DOI:** 10.3390/biom16101496

**Published:** 2026-10-14

**Authors:** Dimitris Spyridakos, Ioannis Zyganitidis, Sofia Petsangouraki, Chrystalla Konstantinou, Alexandros Tsimpolis, Sygkliti-Henrietta Pelidou, Kostas Theodorakis, Michael Malamas, Domna Karagogeos

**Affiliations:** 1 Department of Basic Science, School of Medicine, University of Crete, 71003 Heraklion, Greece; giannisziganitidis@gmail.com (I.Z.); s.petsangouraki@imbb.forth.gr (S.P.); chrys_konstantinou@imbb.forth.gr (C.K.); altsimpolis@hotmail.com (A.T.); kostas.theodorakis@imbb.forth.gr (K.T.); 2 Institute of Molecular Biology and Biotechnology, Foundation for Research and Technology, 70013 Heraklion, Greece; 3 Department of Neurology, School of Medicine, University of Ioannina, 45110 Ioannina, Greece; epelidou@uoi.gr; 4 Center for Drug Discovery, Departments of Chemistry and Chemical Biology and Pharmaceutical Sciences, Northeastern University, Boston, MA 02115, USA; malamas.michael@gmail.com

**Keywords:** excitotoxicity, AMPA, endocannabinoid system, 2-AG, MAGL, ABHD6, neuroprotection, neuroinflammation, microglia, oligodendrocytes

## Abstract

Excitotoxicity mediated by the α-amino-3-hydroxy-5-methyl-4 isoxazolepropionic acid receptor (AMPAR) is a major contributor to neuronal degeneration and inflammation in various neurological disorders. The endocannabinoid system (ECS), specifically through the endocannabinoid 2-arachidonoylglycerol (2-AG), exerts neuroprotective and anti-inflammatory effects, but these actions are limited by enzymatic degradation of 2-AG by monoacylglycerol lipase (MAGL) and α/β-hydrolase domain-containing protein 6 (ABHD6). In the present study we investigated the effects of AM11920, a dual MAGL/ABHD6 inhibitor, in a mouse model of AMPA-induced cortical excitotoxicity and in primary cultures of microglia, neurons, and oligodendrocyte precursor cells (OPCs). Intraperitoneal administration (i.p.) of AM11920 reduced microglial and astrocytic reactivity, attenuated neuronal loss, and increased the density of mature oligodendrocytes at the lesion site in vivo. Importantly, chronic administration of AM11920 in vivo did not affect cannabinoid receptor type 1 (CB1R) protein expression. In vitro, AM11920 suppressed microglial activation by reducing CD68 expression and attenuated interleukin-6 (IL-6) expression, while also protecting primary neurons and OPCs from AMPA-induced cell death via a CB1R-dependent mechanism. Collectively, these findings demonstrate that enhancing 2-AG signaling through dual MAGL/ABHD6 blockade provides neuroprotective and immunomodulatory effects and represents a promising therapeutic strategy against excitotoxic-induced neurodegeneration.

## 1. Introduction

Glutamate is the primary excitatory neurotransmitter in the central nervous system (CNS) that mediates excitatory synaptic transmission [1]. Tight regulation of glutamatergic signaling is essential for normal CNS function, as excessive extracellular glutamate can trigger excitotoxicity, a pathological process that leads to irreversible neuronal injury and death [2]. Overactivation of ionotropic glutamate receptors results in sustained calcium influx, initiating a cascade of deleterious events including mitochondrial dysfunction, oxidative stress, activation of apoptotic pathways and ultimately neuronal degeneration [3,4,5]. Intracellular calcium overload is considered the central driver of excitotoxicity, acting as the key trigger for multiple downstream mechanisms that culminate in cellular dysfunction and death [6]. Although N-methyl-D-aspartate receptors (NMDARs) were initially regarded as the primary route of pathological calcium entry, subsequent studies demonstrated that α-amino-3-hydroxy-5-methyl-4-isoxazolepropionic acid receptors (AMPARs), which mediate fast excitatory synaptic transmission, also play a critical role in excitotoxic injury [7]. A certain subset of AMPARs, lacking the GluA2 subunit, are fully permeable to calcium ions and capable of mediating the deleterious effects of excitotoxicity in neuronal survival [8,9]. Emerging evidence suggests that AMPAR-mediated excitotoxicity is a central mechanism underlying neurodegeneration across a broad spectrum of neurological disorders, including ischemia, epilepsy, traumatic brain injury (TBI), multiple sclerosis, Parkinson’s, Alzheimer’s disease (AD) and others [7].

Beyond its direct toxic effects on neurons, excitotoxicity is a potent driver of neuro-inflammation, eliciting robust activation of both microglia and astrocytes that further amplifies inflammatory responses and exacerbates tissue damage [10,11]. In response to excitotoxic stimuli, microglia rapidly undergo morphological and functional changes associated with increased proliferation, migration to the lesion and increased inflammatory activity [12]. Activated microglial cells release several pro-inflammatory cytokines, such as tumor necrosis factor α (TNFα), interleukin-6 (IL-6) and interleukin-1β (IL-1β), which exacerbate neuronal dysfunction and accelerate degeneration [13,14,15,16]. Similarly, astrocytes also become activated following excitotoxic injury by upregulating the expression of glial fibrillary acidic protein and acquiring a pro-inflammatory phenotype, which potentiates inflammation [17,18]. Importantly, astrocytes are the primary regulators of extracellular glutamate clearance and metabolism within the CNS. Under pathological conditions, their ability to maintain glutamate homeostasis becomes compromised, resulting in impaired glutamate uptake, elevated extracellular glutamate concentrations, and further amplification of excitotoxic damage [19]. Thus, excitotoxicity and neuro-inflammation form a self-reinforcing pathological cycle that contributes substantially to progressive neuronal loss in a wide range of neurological disorders.

The ECS consists primarily of the cannabinoid receptor type 1 and 2 (CB1R and CB2R), the endogenous lipid ligands anandamide (AEA) and 2-arachidonoylglycerol (2-AG), as well as the enzymes responsible for their synthesis and degradation [20]. Among the endocannabinoids, 2-AG is the most abundant in the brain and functions as a full agonist at both CB1R and CB2R, making it a principal mediator of endocannabinoid signaling [21]. Due to its widespread distribution and diverse physiological functions, the ECS has emerged as a critical regulator of neuronal excitability, neuro-inflammation and cell survival. Activation of CB1R, which is highly expressed in neurons, has been shown to reduce presynaptic release of glutamate at the excitatory synapses and attenuate glutamatergic signaling [22,23]. In contrast, CB2R is predominantly associated with immune and glial cells, where it plays a central role in regulating inflammatory and immune responses within the CNS [24]. A growing body of evidence demonstrates that activation of cannabinoid signaling pathways has been shown to mitigate neuronal injury, reduce neuro-inflammatory responses, and promote cellular survival, highlighting the ECS as a promising therapeutic target for disorders characterized by excitotoxicity and chronic neuro-inflammation [25,26].

Biological actions of 2-AG are tightly regulated by enzymatic degradation. Approximately 85% of brain 2-AG hydrolysis is mediated by monoacylglycerol lipase (MAGL) establishing it as the primary regulator of endocannabinoid tone [27]. The α/β-hydrolase domain-containing 6 (ABHD6) protein contributes to a smaller, yet physiologically significant, fraction of 2-AG metabolism and plays an important role in local endocannabinoid signaling [28]. Pharmacological inhibition of either MAGL or ABHD6 elevates endogenous 2-AG levels and affords neuroprotection and anti-inflammatory actions in various animal models of neurological disease [29,30,31]. Among the available pharmacological treatments, dual inhibition of MAGL and ABHD6 may provide a more comprehensive and sustained enhancement of endogenous 2-AG signaling than selective inhibition of either target alone. AM11920 is a potent dual inhibitor of MAGL and ABHD6, previously shown to elevate endogenous 2-AG levels and attenuate neuronal loss and glial activation in the context of AMPA-induced retinal excitotoxicity [32,33].

However, whether AM11920 exerts similar neuroprotective effects in models of cortical excitotoxicity remains unknown and warrants further investigation. In the present study, we investigated the therapeutic potential of AM11920, a dual MAGL/ABHD6 inhibitor, in a mouse model of AMPA-induced cortical excitotoxicity in vivo and in primary cultures of neurons, microglia and oligodendrocyte precursor cells (OPCs). Specifically, we examined whether pharmacological enhancement of endogenous 2-AG signaling could mitigate excitotoxic neuronal injury and attenuate neuro-inflammation. Our findings clearly demonstrate that AM11920 exerts considerable neuroprotective and immunomodulatory effects, significantly reducing excitotoxic damage while attenuating inflammatory processes. Collectively, these findings identify dual MAGL/ABHD6 inhibition as a promising therapeutic strategy for limiting excitotoxic neurodegeneration and underscore the potential of targeting 2-AG metabolism in neurological disorders characterized by excitotoxicity and neuro-inflammation.

## 2. Materials and Methods

### 2.1. Animals

Eight-week-old male C57BL/6 mice, weighing approximately 25–30 g, were employed in the in vivo experiments. Animals were housed 2–3 per cage in the animal facility of the Institute of Molecular Biology and Biotechnology (IMBB)—Foundation for Research and Technology Hellas (FORTH) under stable conditions in a 12 h light–dark cycle and with food and water provided ad libitum. The detailed experimental protocol was licensed by the Veterinary Council (license numbers: EL91-BIObr-01 and EL91-BIOexp-02). All experimental procedures were performed by skilled individuals, holders of FELASA certificate, and were conducted, taking into consideration animal well-being and reduction in suffering, in accordance with the 3R principles, the EU directives (2010/63/EU and L 276/33/20.10.2010) and the Greek legislation (Presidential Decree 56/30.04.2013) for the care and use of laboratory animals. Euthanasia was performed by anesthetic overdose with a ketamine/xylazine mixture and/or cervical dislocation.

### 2.2. Drugs and Reagents

(R,S)-α-Amino-3-hydroxy-5-methyl-4-isoxazolepropionic acid hydrobromide (AMPA, Tocris, Bristol, UK) was dissolved at a stock concentration of 10 mM in water for injection (wfi) and further diluted to the working concentration of 5 mM in sodium chloride 0.9% solution (NaCl 0.9%). The dual MAGL/ABHD6 inhibitor, AM11920, and the CB1R antagonist, SR141716 (kindly offered by Prof. M.S. Malamas) were dissolved in dimethyl sulfoxide (DMSO, AppliChem GmbH, Darmstadt, Germany) at a stock concentration of 10 mM. For in vivo experiments, AM11920 was further diluted in NaCl 0.9% at a concentration of 0.5 mg/kg. For in vitro experiments, all agents were diluted to their final concentrations in the respective culture media.

### 2.3. In Vivo Mouse Model of AMPA Excitotoxicity

Anesthesia was induced in mice by intraperitoneal injection (i.p.) of 5 μL/per g of bodyweight of a ketamine/xylazine/NaCl 0.9% mixture (150 μL ketamine/100 μL xylazine/450 μL NaCl 0.9%). After achieving a surgical plane of anesthesia, mice were carefully mounted on the stereotaxic apparatus. Stock AMPA (10 mM) was diluted in NaCl 0.9% at a concentration of 5 mM and injected with the use of microinjection pump (CMA Microdialysis, Kista, Sweden) into the M2 region of the prefrontal cortex up to layer V [anteroposterior axis (AP): +1 mm from bregma, mediolateral axis (ML): −1 mm from bregma, dorsoventral axis (DV): −1.5 mm from the dura mater] [34]. The experimental group received an injection of AMPA solution (5 mM) at a volume of 1 μL and a flow rate of 0.1 μL/min, while the control group received an equal volume of vehicle solution, consisting of 50% wfi and 50% NaCl 0.9% (*v*/*v*). Animals were sutured and left to fully recover on a heating pad and then were returned to their home cages.

Treatment with AM11920 began immediately after animals had fully recovered. Briefly, stock AM11920 (10 mM), dissolved in DMSO, was diluted in 0.9% NaCl until it reached a working concentration of 0.5 mg/kg and was injected i.p. to the animal once daily for seven consecutive days. The control group received an equal amount of the vehicle solution (NaCl 0.9% and DMSO). Although MAGL inhibitors are typically administered at higher doses above 1 mg/kg [35], here we employed a lower dose (0.5 mg/kg) to achieve sufficient modulation of 2-AG, while simultaneously avoiding cannabinoid adverse effects and CB1R desensitization. This dose was chosen based on the study of [33], where it is shown that i.p. administration of AM11920 elevates 2-AG levels in rat retina and provides neuroprotection without affecting CB1R expression. Twenty-four hours after the last i.p. administration of AM11920, animals were transcardially perfused with Phosphate-Buffered Saline 1X (PBS 1X), followed by paraformaldehyde 4% (PFA 4%) solution, and their brains were extracted. A different group of animals were euthanized by cervical dislocation. Their brains were extracted and rapidly dissected on ice to isolate the lesion area of the prefrontal cortex. A 6 mm^2^ area of the prefrontal cortex at the injection site was dissected and stored at −80 °C for conducting Western blot analysis.

### 2.4. Tissue Preparation

Fixed brains were post-fixed by immersion in 4% PFA (Sigma-Aldrich, Darmstadt, Germany) for an additional 30 min, then transferred into a 30% sucrose solution for cryoprotection. Cryoprotected tissues were embedded in a gelatin (7.5% *w*/*v*, Sigma-Aldrich, Darmstadt, Germany) and sucrose (15% *w*/*v*) solution. Polymerized trapezoids containing the brain sample were then snap frozen in −45 to −35 °C on dry ice and stored at −80 °C. Frozen samples were then cryosectioned at 15 μm thickness on a cryotome and mounted on Superfrost Plus glass slides (Thermo Fisher Scientific, Waltham, MA, USA). For each sample, ten slides were taken, with each containing eight to ten coronal sections, which include the lesion area of the prefrontal cortex. Slides were stored in −20 °C until further processing.

### 2.5. Immunohistochemistry

Slides with the embedded cryosections were post-fixed by immersion in ice-cold acetone at −20 °C for 10 min. Following post-fixation, sections were washed in 1X PBS 3 × 5 min. Afterwards, sections were incubated in blocking solution consisting of 5% bovine serum albumin (fraction V, BSA, AppliChem, GmbH, Darmstadt, Germany, Cat# A1391) and 0.5% TritonX-100 in 1X PBS, for 1 h at room temperature. After blocking, sections were incubated with the appropriate primary antibodies, diluted in the blocking solution, at 4 °C overnight. The following primary antibodies were used: An anti-ionized calcium-binding adapter molecule 1 (anti-Iba1, rabbit polyclonal, 1/500, Invitrogen, Cat# PA5-21274, Carlsbad, CA, USA) was employed for microglia recognition, while for astrocytes we chose an antibody against glial fibrillary acidic protein (anti-GFAP, mouse monoclonal, 1/2000, Sigma-Aldrich, Cat# G3893). An anti-neuronal nuclei antibody (anti-NeuN, rabbit polyclonal, 1/500, Cat# 24307, Cell Signaling Technology, Danvers, MA, USA) was used to stain neuronal cells, and an anti-myelin basic protein (anti-MBP, chicken polyclonal, 1/1000, Abcam, Cat# ab123499, Cambridge, UK) to visualize myelin. Finally, mature oligodendrocytes (Ols) were stained against adenomatous polyposis coli factor (anti-APC, clone CC1, mouse monoclonal, 1/100, Calbiochem, Cat# OP80-100UG, San Diego, CA, USA) while OPC were identified by labeling with the anti-platelet-derived growth factor alpha antibody, (PDGFRα, mouse monoclonal, 1/200, R&D AF1062, Minneapolis, MN, USA). Following primary antibody incubation, sections were washed 3 × 5 min in 1X PBS and incubated in the secondary antibodies diluted in blocking solution for 1.5 h at room temperature. The following fluorophore-conjugated secondary antibodies were employed: anti-chicken (rabbit, 1/800, CF543, Biotium, Cat#20312, Fremont, CA, USA), anti-rabbit (goat, 1/800, CF 488A, Biotium, Cat# 20012, USA), anti-mouse (goat, 1/800, Alexa Fluor 555, Thermo Fisher Scientific, Cat# A-21422, USA), anti-mouse (goat, 1/800, Alexa Fluor 647, Abcam, Cat# ab150119, USA), anti-goat (donkey, 1/800, Alexa Fluor 647, Abcam, Cat# 1b150131, USA). 4′,6-Diamidino-2-phenylindole (DAPI) (1/1500, Invitrogen, Cat# MP01306, USA) was also added to the secondary antibody buffer for visualizing cell nuclei. Afterwards, slides were mounted in Mowiol 4-88 Reagent mounting medium (Merck Millipore, Cat# 475904, Darmstadt, Germany) and coverslipped.

### 2.6. Western Blot

Prefrontal cortices from C57BL/6 mice, were thawed on ice and sonicated at 50 Hz in 200 µL ice-cold radio-immunoprecipitation assay buffer (RIPA) buffer [50 mM Tris-HCl pH 8.0, 150 mM NaCl, 1% Triton X-100, 0.5% sodium deoxycholate (DOC)] with protease inhibitor cocktail (Sigma-Aldrich, Cat# P8340). Afterwards, samples were kept on ice for 20 min and then were centrifuged 2 × 20 min at 12,000 rpm at 4 °C. Total protein concentration in each sample was defined by Bradford assay (Bio-Rad Laboratories, Inc., Hercules, CA, USA, Cat# 5000006). Subsequently, 4X Laemmli buffer and 0.1 M dithiothreitol (DTT) (Sigma-Aldrich, Cat# D-9779) were added to the samples and were then heated at 95 °C for 5 min. Proteins were separated by sodium dodecyl sulfate polyacrylamide gel electrophoresis (SDS-PAGE) in a gel containing 10% polyacrylamide. Following separation, proteins were transferred to a nitrocellulose membrane (Sigma-Aldrich, Cat# 10600002) for 1.5 h at 320 mA on ice. The membrane with the transferred proteins was washed in PBST (PBS 1X-Tween-20 0.1%), followed by incubation in blocking solution [3% BSA AppliChem, GmbH, Darmstadt, Germany, Cat# A1391) in PBST] for 1 h at room temperature and then incubation with a primary antibody against cannabinoid receptor 1 (anti-CB1R, rabbit polyclonal,1:1000, Cayman, Cat#10006590, Ann Arbor, MI, USA) overnight at 4 °C. Thereafter membranes were washed 3 × 15 min in PBST and incubated in a horseradish peroxidase conjugated antibody (HRP-goat anti-rabbit IgG, 1/5000, Invitrogen, USA). Eventually Blots were developed using chemiluminescence (ImmobilonClassico Western HRP substrate, Cat# WBLUC0500, Merck Millipore). Intensity of bands was quantified by employing Image Lab 6.1 software (Bio-Rad Laboratories, Inc.).

### 2.7. Quantitative Real timePCR 

Total RNA was extracted from cultures using TRIzol Reagent (Thermo Fisher Scientific, 15596026), and cDNA was synthesized using the High-Capacity cDNA Reverse Transcription kit (Thermo Fisher Scientific, 4368814) according to the supplier’s protocols. For quantitative real time (RT-PCR), we used 1 μL of cDNA and the KAPA SYBR Fast qPCR Master Mix kit (2×) (KAPABiosystems Ltd., London, UK, KK4602) according to the supplier’s instructions. The cycling conditions consisted of an initial denaturation at 95 °C for 20 s, followed by 40 cycles of denaturation at 95 °C for 3 s and annealing/extension at 60 °C for 30 s on a StepOne RT PCR System (Thermo Fisher Scientific). After completion of qPCR, a melt curve of the amplified products was performed, and gene expression was normalized to β-actin. Data were collected and analyzed using the StepOne Software v2.3 (Thermo Fischer Scientific). Mouse primer sequences used are listed in Table 1.

### 2.8. In Vitro Experiments

#### 2.8.1. Primary Microglia and OPC Cultures

Primary mixed glia cultures were generated from the cortices of postnatal day 2 (P2) C57BL/6 mouse pups, following the protocol described in Eigel et al. [36]. After dissociation, cells were cultured in 75 cm^2^ flasks coated with 0.01 mg/mL of poly-D-lysine (PDL, Cat# P7405, Sigma-Aldrich), in 10 mL of DMEM (Dublecco’s Modified Eagle Medium) with GlutaMAXTM, 4.5 g/L d-Glucose, Pyruvate (Gibco, Paisley, UK, Cat# 61965-026) (Ca^2+^ concentration: 1.8 mM), supplemented with 10% FBS and 1% Penicillin/Streptomycin (10,000 units, Gibco, Cat# 15140-122) at 37 °C in 5% CO_2_. Once cultures reached confluence, at 12–14 days in vitro (DIV), microglia and OPCs were isolated and separated through mechanical shaking at an orbital shaker at 37 °C.

For microglial isolation, flasks were shaken at 200 rpm for 1 h at 37 °C, as described in [37]. At DIV0, which is considered the day of their isolation, microglial cells were seeded onto PDL-coated glass coverslips in 24-well plates at a density of 1.5 × 10^5^ cells/well for immunohistochemistry experiments. In addition, for RT-PCR, cells were cultured directly on 24-well plates with no glass coverslips applied. At DIV2, microglia were pretreated with AM11920 at a final concentration of 0.5 and 1 μM. The 1 h pre-treatment range was selected based on in vivo data demonstrating that MAGL inhibition with the JZL184 inhibitor induces the maximum elevation in endogenous 2-AG levels at 1 h after treatment [38]. Then, 1 h later, lipopolysaccharide (LPS) was applied to the cells at 100 ng/mL to induce their classical activation, while AM11920 was also replenished in the medium. Control cells received a medium with the same amount of DMSO as AM11920-treated cells. For immunocytochemistry experiments, microglial cells were fixed 24 h after LPS and AM11920 co-administration, while for RT-PCR experiments, microglial cells were lysed and processed for RNA extraction 4 h after LPS treatment.

Following microglia isolation, flasks were additionally shaken at 250 rpm for 16 h, at 37 °C, a process which allows the separation of OPCs from the remaining astrocytes, as described in [39]. OPCs were seeded at a density of 6 × 10^4^ cells/well in PDL-coated 9 mm glass coverslips in 48 well plates. They were cultured in a medium made of DMEM (with GlutaMAX^TM^, 4.5 g/L d-Glucose, Pyruvate from Gibco, Cat# 61965-026), supplemented with 1% N2 supplement (Thermo Fisher Scientific, Cat#17502), 100 ng/mL D-biotin (Sigma-Aldrich, Cat# B4501), 1% BSA (Sigma-Aldrich, A6003), 60 μg/mL N-acetylcysteine (Sigma-Aldrich, Cat# A8199) and 1% penicillin streptomycin (10,000 units, Gibco, Cat# 15140-122). The medium also contained 10 ng/mL platelet-derived growth factor α (PDGFα, Proteintech Cat No. HZ-1215, IL, USA) and 10 ng/mL fibroblast growth factor (FGF, Proteintech Cat No. HZ-1103-GMP) to initiate proliferation of OPCs. OPCs at DIV2, 1 h before administration of 150 μM AMPA to induce excitotoxicity, were pretreated with AM11920 at four different concentrations (0.5, 1, 5 and 10 μM). AM11920 was also replenished and co-administered with AMPA. Twenty-four h after AMPA and AM11920 co-treatment, OPCs were fixed in 4% PFA and processed for immunocytochemistry.

#### 2.8.2. Primary Neuronal Cultures

Primary neuronal cell cultures were prepared from the cortices of embryonic day 16.5 (E 16.5) C57BL/6 mouse embryos of both sexes. Pregnant female mice were euthanized by cervical dislocation, after which embryos were extracted and transferred into Petri dishes containing ice-cold 1X Hank’s Balanced Salt Solution without CaCl_2_/MgCl_2_ (HBSS-/-, Gibco, Cat# 14065-049). Following extraction of embryonic brains, cortices were dissected in ice-cold 1X HBSS-/-, the meninges were removed, and cortices were transferred in ice-cold 1X HBSS-/-, supplemented with 0.1 mg/mL DNase (Sigma-Aldrich, Cat# DN25). Afterwards, cortices were washed three times with ice-cold 1X HBSS-/- and incubated 1× HBSS-/-, containing 2.5% trypsin (Gibco, Cat# 15090-046) and 10 mg/mL DNase (Sigma-Aldrich, Cat# DN25) for 15 min at 37 °C to induce cell dissociation. Trypsin was deactivated by performing three washes with prewarmed DMEM (GlutaMAX^TM^, 4.5 g/L d-Glucose, Pyruvate, Gibco, Cat# 61965-026), supplemented with 30% FBS (Gibco, Cat# 10437028). Subsequently, DMEM and FBS were removed and replaced with plating medium, consisting of Neurobasal Plus (Gibco, Cat# A35829-01), B27 (50× Gibco, Cat# 17504), N2 supplement (100× Gibco, Cat#17502-048), Penicillin/Streptomycin 10,000 units (Gibco, Cat# 15140-122), L-Glutamine (200 mM Gibco, Cat# 25030024), and DNase (0.1 mg/mL Sigma-Aldrich, Cat# DN25) and cortices were triturated. Following trituration, dissociated cortices were centrifuged at 500 g for 5 min at 4 °C, the supernatant was removed and the pellet was resuspended in plating medium. To remove large undissociated pieces, cell suspension was passed through a 70 μm cell strainer, and eventually cells were seeded at a density of 3 × 10^5^ cells/well on PDL-coated 13 mm coverslips in 24-well plates in plating medium. Cells were incubated at 37 °C with 5% CO_2_ until DIV21, when the treatment began.

At DIV21, neuronal cultures were pretreated with AM11920 at 2 different concentrations (0.5 and 1 μM). In a separate group, cells were treated with the CB1R antagonist SR141716 (2 μM) 30 min before the pre-treatment with AM11920. Subsequently, 1 h after the pre-treatment with AM11920, AMPA (150 μM) was added to the cells, while AM11920 (0.5 and 1 μM) was also replenished. Cells were subjected to AMPA for 1 h and then were removed and replaced by conditioned medium that was collected from the cells prior to the initiation of the experiment. Eventually, 24 h after AMPA administration, at DIV22, cells were fixed in 4% PFA and processed for immunocytochemistry.

### 2.9. Immunocytochemistry

Fixed coverslips with microglia, OPCs, and neurons were washed 3 × 5 min with 1X PBS. Afterwards, cells were incubated in blocking buffer, consisting of 1% BSA and 0.1% Triton-100 for 30 min at room temperature. Subsequently, cells were incubated in the appropriate primary antibodies diluted in the blocking solution for 1 h at room temperature. More specifically, microglial cells were stained against Iba1 (rabbit polyclonal, 1/500, Invitrogen, Cat# PA5-21274, USA), which served as a pan microglial marker and against cluster of differentiation 68 factor (CD68, rat monoclonal, 1/200, Biolegend, Cat# 137001, San Diego, CA, USA), a marker typically upregulated in reactive-phagocytic microglia. Neurons were stained with an antibody against Neuronal nuclei marker (NeuN, rabbit polyclonal, 1/500, Cell Signaling Technology, Cat# 24307), while an anti-microtubule-associated protein 2 antibody (anti-MAP2, mouse monoclonal, 1/400, Sigma-Aldrich, Cat# MAB3418) stained the neuronal arborization. An antibody against PDGFRα (rat monoclonal, 1/100, Merck Millipore, Cat# CBL1366) was employed for the recognition of OPCs. Cleaved caspase 3 (cl. Casp. 3, rabbit monoclonal, 1/500, Cell Signaling Technology, Cat# 9464S) served as a marker of apoptotic cell death for OPCs and neurons. Following primary antibody incubation, cells were washed with three instant washes in 1X PBS and incubated in the secondary fluorescent antibodies. The following secondary antibodies were used: anti-rabbit (goat, 1/800, CF 488A, Biotium, Cat# 20012), anti-rabbit (goat, 1/800, CY3, Novus Biologicals, Cat# 6939, Centennial, CO, USA), anti-mouse (goat, 1/800, Alexa Fluor 555, Thermo Fisher Scientific, Cat# A-21422), anti-mouse (goat, 1/800, Alexa Fluor 647, Abcam, Cat# ab150119), anti-rat (goat, 1/800, CF 488A, Biotium, Cat# 20023). All cells were also stained with DAPI (1/1500, Invitrogen, Cat# MP01306), thus enabling nuclei visualization. Eventually, coverslips were mounted on glass slides by applying Mowiol 4-88 Reagent mounting medium (Merck Millipore, Cat# 475904).

### 2.10. Microscopy and Image Quantification

Images were obtained on a confocal laser scanning microscope (Leica microsystems, TCS SP8, Wetzlar, Germany). In the in vivo experiments, photomicrographs were obtained at 10× magnification, 2.5× zoom, 400 scan speed, and 1024 × 1024 pixels, with a z-step size of 1.5 μm. For in vitro experiments all parameters remained the same, except for magnification, which was set at 40× and zoom at 2. All parameters, including laser intensity, detector offset and gain, remained constant across samples from the same experiment. Images were analyzed by employing Fiji ImageJ software, version 1.54g (National Institutes of Health, Bethesda, MD, USA).

In the in vivo experiments, each separate biological replicate (*n*) results from the average of three sections that were measured. Densitometric analysis of Iba1 and CC1 immunoreactivity represents the total number of Iba1+ and CC1+ cells, respectively, to the area measured in mm^2^. For NeuN, the necrotic area (mm^2^) where NeuN immunoreactivity was absent was calculated. In GFAP and MBP experiments, fluorescence intensity was quantified as the mean gray value, representing the intensity within the specific region of interest (ROI). In the in vitro experiments, each biological replicate consists of three different coverslips, with three tile scans each; each tile scan contains six tiles. Each biological replicate comes from a different litter of pups. Quantification of microglial density in vitro was the same as the in vivo, referenced above. Density of Iba1+ primary microglial cells, PDGFRα+, cl. casp.3+ primary OPCs, and NeuN+ primary neurons is expressed as the total number of each cell type to the area (mm^2^) measured. For CD68 and MAP2 immunocytochemistry, the mean gray value was employed for the quantification. Both at the in vivo and at the in vitro experiments, analysis was performed in the max projection output of each photomicrograph.

### 2.11. Statistical Analysis

Quantitative data are presented in a scatter dot plot with bar graphs format and expressed as mean ± standard error of the mean (SEM). Statistical analysis and formation of graphs were performed in GraphPad Prism 8 software (GraphPad Software, San Diego, CA, USA). Comparisons among groups were conducted by employing one-way analysis of variance (ANOVA), followed by Tukey’s test for multiple comparisons. Differences were considered statistically significant if *p* < 0.05.

## 3. Results

### 3.1. AM11920 Reduces Microglial and Astrocytic Density In Vivo

**Figure 1:**
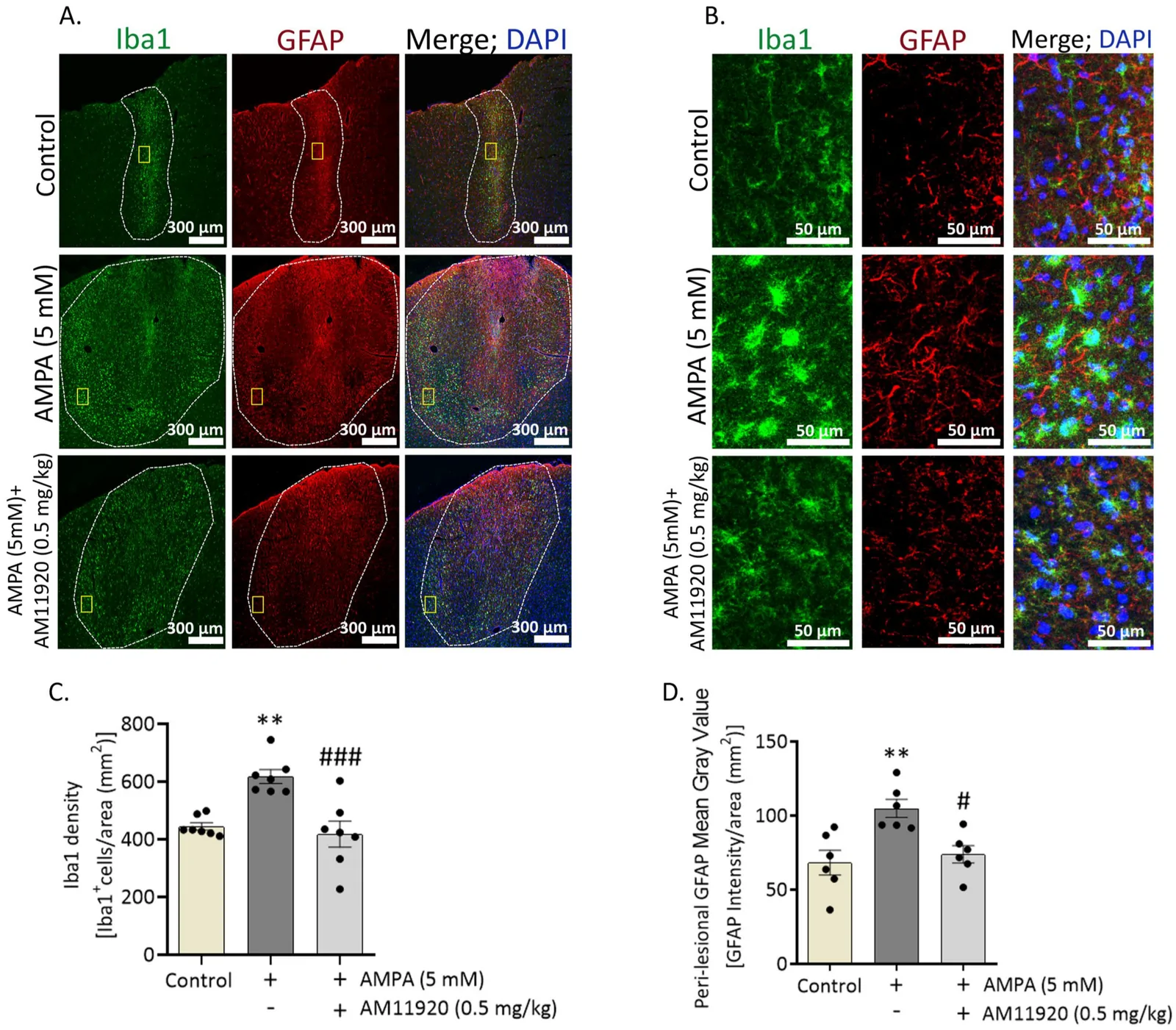
AM11920 reduces microglial and astrocytic density in vivo. (**A**) Representative photomicrographs of the M2 region of the prefrontal cortex in Control, AMPA (5 mM), and AMPA + AM11920 (0.5 mg/kg), stained for Iba1 (green), GFAP (red), and DAPI (blue). White dashed line marks the lesion site. Scale bar: 300 μm. (**B**) Higher magnification of the area outlined in the yellow rectangle. Scale bar: 50 μm. (**C**,**D**) Quantification analysis of Iba1 density (**C**) and GFAP immunoreactivity (**D**) in Control (*n* = 6), AMPA (*n* = 6) and AMPA + AM11920 (*n* = 6)-treated animals. Data are expressed as mean ± SEM. One-way ANOVA was used for statistical comparison, followed by Tukey’s post hoc analysis for multiple comparisons. ** *p* < 0.01, compared to Control; # *p* < 0.05, ### *p* < 0.001 compared to AMPA.

Microglial and astrocytic activation are key elements, present early, in the pathophysiological events triggered by an excitotoxic insult [40]. Although glial activation can initially serve protective functions, prolonged activation contributes to disease progression through the maintenance of a chronic pro-inflammatory state [41]. Here we employed Iba1 as a marker for microglia and GFAP as a marker for astrocytes to examine their response after applying an excitotoxic stimulus, as well as the effect of AM11920 on these cells. AMPA injection to the cortex induced a clear morphological transformation with hypertrophic cell bodies, compared to control cells (Figure 1A,B) indicative of enhanced reactivity and a strong increase in microglial density (617.7 ± 63.73 cells/mm^2^, ** *p* = 0.0021, compared to Control) (Figure 1C) in a greater area around the injection site. At the core of the lesion, necrotic tissue was evident, with minimal or no microglial cells at all (Figure 1A,B). For this reason, microglial density was quantified in the peri-lesion area, defined as the area around the necrotic core with visible Iba1 reactivity. Treatment with AM11920 effectively suppressed the AMPA-induced elevated microglial density (417.3 ± 118.4 cells/mm^2^, ### *p* = 0.0021, compared to AMPA; *p* > 0.05, compared to Control) (Figure 1C), which was accompanied by a return of their morphological features to a morphology similar to that of Control (Figure 1B), indicating a reduced reactivity status. Microglial cell density in control animals was 444.5 ± 34.17 cells/mm^2^ (Figure 1C). Together, these findings indicate that AM11920 attenuates both the proliferative and morphological hallmarks of microglial activation following excitotoxic injury.

Astrocytic reactivity was assessed by GFAP immunoreactivity in the peri-lesional cortex. Control animals that received intracerebral injection of vehicle into their cortex displayed dense GFAP immunoreactivity, as shown by the mean gray value (48.18 ± 8.235) restricted near the area of the injection site (Figure 1A,B). In contrast, AMPA-treated animals exhibited extensive GFAP immunoreactivity, which covered a much larger area around the injection site (26.82 ± 10.05) (Figure 1A). Here, we also proceeded to quantify the peri-lesion area, as necrotic tissue at the core of the lesion was also present (Figure 1A,B). One-way ANOVA revealed a significant difference among the three groups [F(2,18) = 13.87, *** *p* = 0.0002] (Figure 1D). Multiple comparisons showcased that the mean gray value of GFAP immunoreactivity was clearly upregulated in the AMPA-treated animals compared to the Control group (*** *p* = 0.0003, compared to Control) (Figure 1D). Administration of AM11920 completely prevented the effect of AMPA in upregulating GFAP mean gray value (44.96 ± 5.649, ## *p* = 0.0017, compared to AMPA; *p* > 0.05, compared to Control), suggesting that the inhibitor can reduce astrocytic reactivity.

### 3.2. AM11920 Attenuates AMPA-Induced Neuronal Death In Vivo, Without Affecting CB1R Protein Expression

**Figure 2:**
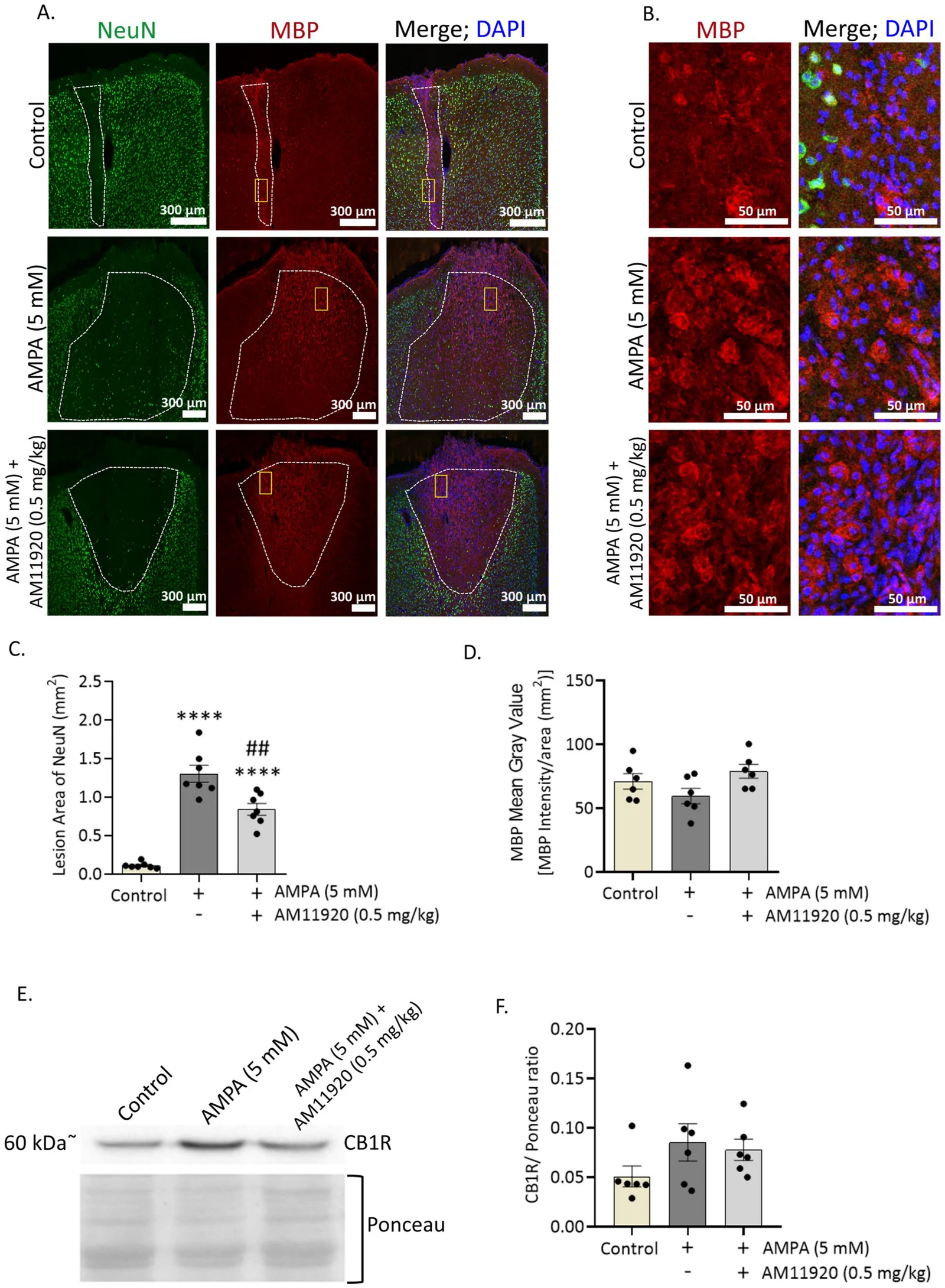
AM11920 attenuates AMPA-induced neuronal death in vivo, without affecting CB1R protein expression. (**A**) Representative photomicrographs of the M2 region of the prefrontal cortex of Control, AMPA (5 mM), and AMPA + AM11920 (0.5 mg/kg), stained for NeuN (green), MBP (red), and DAPI (blue). White dashed line marks the lesion site. Scale bar: 300 μm. (**B**) Higher magnification of the MBP-stained area outlined in the yellow rectangle shown in (**A**). Scale bar: 50 μm. (**C**,**D**) Quantification analysis of the area outlined in dashed white line, lacking NeuN+ nucleii (**C**) and MBP immunoreactivity (**D**) in Control (*n* = 6), AMPA (*n* = 6) and AMPA + AM11920 (*n* = 6)-treated animals. (**E**) Representative immunoblot for CB1R expression at the lesion site, in Control, AMPA and AMPA + AM11920 (0.5 mg/kg)-treated animals. The original Western blot images are in the Supplementary Figure S1. (**F**) Quantification analysis of CB1R expression at the lesion site in Control (*n* = 6), AMPA (*n* = 6) and AMPA + AM11920 (*n* = 6)-treated animals. Data are expressed as mean ± SEM. One-way ANOVA was used for statistical comparison, followed by Tukey’s post hoc analysis for multiple comparisons. **** *p* < 0.0001, compared to Control; ## *p* < 0.01, compared to AMPA.

Glutamate-mediated excitotoxicity is a well-established driver of neuronal injury and cell death. Neuronal loss here was estimated by performing immunohistochemistry against NeuN, a marker present in neuronal nuclei, and by measuring the necrotic area with no NeuN+ nuclei evident. Control animals displayed a small necrotic area near the site of the vehicle injection in the prefrontal cortex (0.1121 ± 0.039) (Figure 2A,C). One-way ANOVA revealed a significant effect of both AMPA and AM11920 treatment [F(2,18) = 58.45, **** *p* < 0.0001] (Figure 2C). AMPA treatment significantly increased the magnitude of the necrotic area (1.303 ± 0.293, #### *p* < 0.0001, compared to Control) (Figure 2C), indicating increased neuronal loss. AM11920 treatment managed to partially obstruct the effect of AMPA (0.8408 ± 0.206, ## *p* = 0.0016, compared to AMPA; **** *p* < 0.0001, compared to Control) (Figure 2C), suggesting a neuroprotective property.

Given the fact that oligodendrocytes, which are responsible for myelin formation, are sensitive to excitotoxic insults [42], we proceeded to evaluate the effect of AMPA on myelin expression by employing an antibody against MBP, a protein highly expressed in myelin. However, one-way ANOVA did not reveal any significant effect of AMPA or AM11920 treatment on MBP expression [F(2,15) = 2.742, *p* > 0.05] (Figure 2D). Mean gray value of Control MBP immunoreactivity was 70.99 ± 14.99, while AMPA did not significantly alter it (59.47 ± 14.88, *p* > 0.05, compared to Control) (Figure 2A,B,D). Similarly, AM11920 treatment had no significant effect on MBP expression (78.85 ± 13.33, *p* > 0.05 compared to AMPA and Control) (Figure 2A,B,D). These findings suggest that, under the experimental conditions employed, AMPA-induced excitotoxic injury did not produce measurable alterations in MBP expression, despite causing substantial neuronal loss and glial activation.

Previous studies have shown that repeated administration of inhibitors of MAGL activity, which elevates the endocannabinoid tone through increased 2-AG levels, can lead to CB1R desensitization/CB1R downregulation in a dose- and time-dependent manner and can dampen the protective properties mediated by CB1R activation [43,44]. To determine whether prolonged AM11920 administration produced similar effects, CB1R protein expression was assessed by Western blot analysis (Figure 2E,F). Basal CB1R expression in Control was 0.0507 ± 0.026. One-way ANOVA did not reveal any significant effect on CB1R expression [F(2,15) = 1.676, *p* > 0.05] (Figure 2F) of either AMPA (0.0850 ± 0.046) or AM11920 treatment (0.0777 ± 0.027) (Figure 2E,F), indicating that administration of AM11920 for seven consecutive days did not induce alterations on CB1R expression.

### 3.3. AM11920 Upregulates Mature Oligodendrocytes Population at the Lesion Site In Vivo

**Figure 3:**
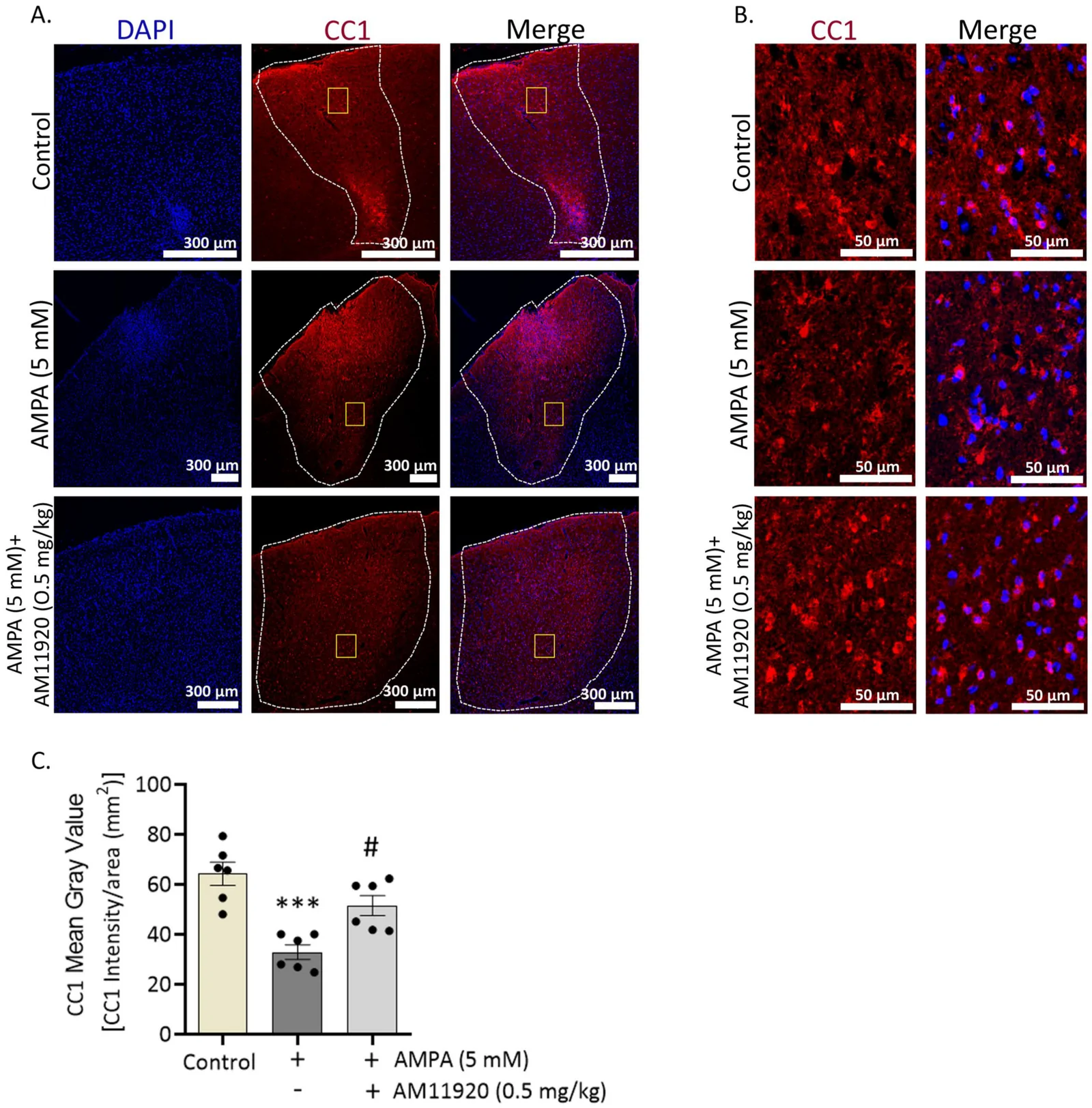
AM11920 upregulates mature oligodendrocyte population at the lesion site in vivo. (**A**) Representative photomicrographs of the M2 region of the prefrontal cortex in Control, AMPA (5 mM), and AMPA + AM11920 (0.5 mg/kg), stained for DAPI (blue) and CC1 (red). White dashed line marks the lesion site. Scale bar: 300 μm. (**B**) Higher magnification of the area outlined in the yellow rectangle. Scale bar: 50 μm. (**C**) Quantification analysis of CC1 density in Control (*n* = 6), AMPA (*n* = 6) and AMPA + AM11920 (*n* = 6)-treated animals. Data are expressed as mean ± SEM. One-way ANOVA was used for statistical comparison, followed by Tukey’s post hoc analysis for multiple comparisons. *** *p* < 0.001, compared to Control; # *p* < 0.05, compared to AMPA.

Although AMPA injection into the prefrontal cortex did not affect MBP expression, we next examined its effect on oligodendrocytes, which, as previously mentioned, are susceptible to AMPA-induced excitotoxicity. For this, we employed CC1, a marker found in mature oligodendrocytes. One-way ANOVA revealed a significant difference among the groups [F(2,15) = 16.40, *** *p* = 0.0002]. Basal levels of CC1 density in Control animals were 240.5 ± 59.23 (Figure 3A,B). Administration of AMPA did not significantly alter CC1 density (283.4 ± 48.99, *p* > 0.05, compared to Control) (Figure 3A,B). In contrast, administration of AM11920 significantly upregulated CC1 density at the lesion site (533.9 ± 82.38, #### *p* < 0.0001, compared to AMPA; **** *p* < 0.0001, compared to Control) (Figure 3A–C), suggesting a potential enhancement of either recruitment or proliferation of oligodendrocytes. PDGFRα was employed as a marker of OPCs. One-way ANOVA indicated a significant effect of treatment in the number of PDGFRα+ OPCs [F(2,15) = 14.04, *** *p* = 0.0004]. Mean population of PDGFRα+ OPCs in Control animals was 47.71 ± 15.92 cell/mm^2^ (Supplementary Figure S2). Administration of AMPA significantly increased the number of PDGFRα+ cells at the lesion site (102.6 ± 19.55, *** *p* = 0.0004, compared to Control) (Supplementary Figure S2A,B), while treatment with AM11920 restored the number of OPCs to Control levels (## *p* = 0.0034, compared to AMPA; *p* > 0.05, compared to Control) (Supplementary Figure S2A,B). Together, these findings indicate that AM11920 enhances OPC differentiation and point to a previously unrecognized effect of AM11920 on oligodendroglial responses following excitotoxic injury.

### 3.4. AM11920 Reduces CD68 Expression and the Expression of the Pro-Inflammatory Cytokine IL-6 in Microglia In Vitro

**Figure 4:**
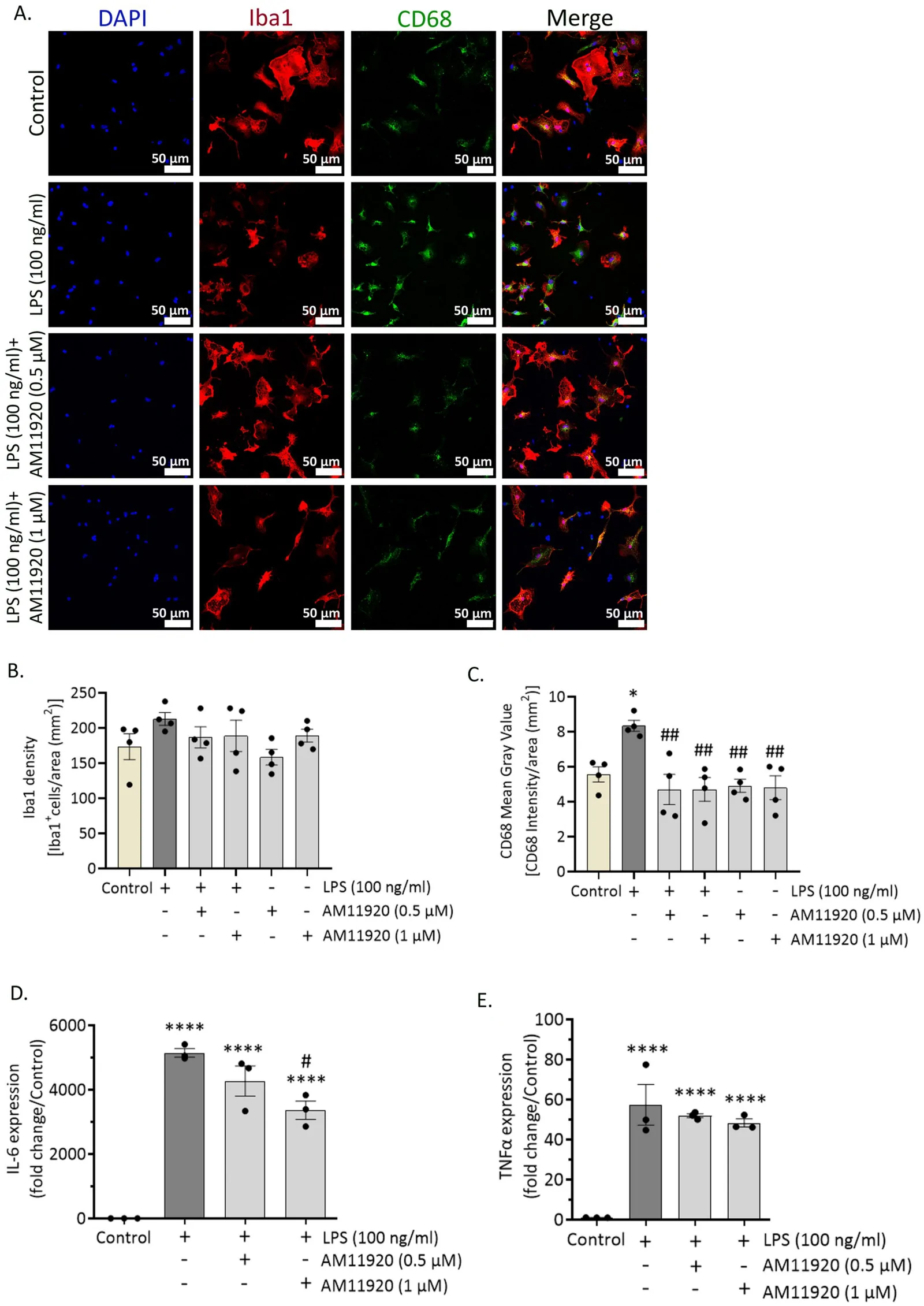
AM11920 reduces CD68 expression and the expression of the pro-inflammatory cytokine IL-6 in microglia in vitro. (**A**) Representative photomicrographs of Control, LPS (100 ng/mL), and LPS + AM11920 (0.5 and 1 μΜ), stained for Iba1 (red), CD68 (green), and DAPI (blue). Scale bar: 50 μm. (**B**,**C**) Quantification analysis of Iba1 density (**B**) and CD68 immunoreactivity (**C**) in Control (*n* = 4), AMPA (*n* = 4), LPS + AM11920 (0.5 μΜ) (*n* = 4), LPS + AM11920 (1 μΜ) (*n* = 4), Control + AM11920 (0.5 μΜ) (*n* = 4) and Control + AM11920 (1 μΜ) (*n* = 4)-treated cells. (**D**,**E**) Quantification analysis of IL-6 (**D**) and TNFα (**E**) gene expression (*n* = 3 for all groups). Data are expressed as mean ± SEM. One-way ANOVA was used for statistical comparison, followed by Tukey’s post hoc analysis for multiple comparisons. * *p* < 0.05, **** *p* < 0.0001 compared to Control; # *p* < 0.05, ## *p* < 0.01, compared to LPS.

To investigate the direct effects of AM11920 on microglial activation, primary mouse microglia were classically activated with LPS to induce a pro-inflammatory phenotype and treated with AM11920. One-way ANOVA of Iba1 densitometric analysis in primary microglial cells revealed no significant differences [F(5,18) = 1.477, *p* > 0.05] (Figure 4A,B) among Control, LPS and AM11920-treated cells (Control: 173.3 ± 36.97; LPS: 212.9 ± 18.09; LPS + AM11920 0.5 μΜ: 186.7 ± 29.96; LPS + AM11920 1 μΜ: 188.8 ± 44.30; AM11920 0.5 μΜ: 158.4 ± 22.04; AM11920 1 μM: 189 ± 18.34) (Figure 4A,B). These results suggest that neither LPS nor AM11920 treatment affects microglial density in vitro.

Microglial cells were also stained for CD68, a lysosomal protein that is upregulated in reactive-phagocytic microglia [45]. One-way ANOVA revealed a significant difference among the groups examined [F(5,18) = 5.818, ** *p* = 0.0023] (Figure 4C). Basal levels of CD68 immunoreactivity in Control microglia were 5.560 ± 0.8596. LPS administration resulted in a significant increase in CD68 mean gray value (8.336 ± 0.6210, * *p* = 0.037, compared to Control) (Figure 4A,C). Co-administration of AM11920 with LPS attenuated the LPS-induced increase in CD68 immunoreactivity, both at the dose of 0.5 μΜ (4.700 ± 1.723, ## *p* = 0.0044, compared to LPS; *p* > 0.05, compared to Control) and at the highest dose of 1 μM (4.703 ± 1.363, ## *p* = 0.0045, compared to LPS; *p* > 0.05, compared to Control) (Figure 4A,C). Administration of AM11920 alone in the absence of LPS did not significantly alter CD68 immunoreactivity compared to Control cells, both at the dose of 0.5 μΜ (4.904 ± 0.7472, *p* > 0.05 compared to Control; ## *p* = 0.0074, compared to LPS) (Figure 4A,C) and at 1 μM (4.797 ± 1.372, *p* > 0.05 compared to Control; ## *p* = 0.0056, compared to LPS) (Figure 4A,C). These results indicate that LPS induces the phagocytic capability of microglia in vitro, while AM11920 tends to suppress this effect without altering basal microglial characteristics. These findings further support the anti-inflammatory and immunomodulatory properties of AM11920 in activated microglia.

Based on the observed effects of AM11920 on microglial activation, we next examined the inflammatory profile of microglial cells treated with LPS and AM11920 by performing RT-PCR for the pro-inflammatory cytokines IL-6 and TNFα. Expression levels for the gene of IL-6 in control microglial cells were 1.0 ± 0.0201. One-way ANOVA displayed a significant effect of treatment in the groups [F(3,8) = 440.5, **** *p* < 0.0001] (Figure 4D). Post hoc analysis for multiple comparisons revealed that LPS administration induced a robust upregulation in the expression levels of IL-6, 4 h after its application (5148 ± 231.2, **** *p* < 0.0001, compared to Control) (Figure 4D), indicating a shift in microglia towards a pro-inflammatory phenotype. Administration of AM11920 partially blocked the LPS-induced upregulation in IL-6 expression at 1 μΜ (3361 ± 488.8, ## *p* = 0.0089, compared to LPS; *** *p* = 0.0001, compared to Control) (Figure 4D), suggesting a suppression of the pro-inflammatory profile, while it had no effect at the dose of 0.5 μM (4274 ± 815.1, *p* > 0.05 compared to LPS, **** *p* < 0.0001, compared to Control) (Figure 4D). Regarding TNFα, its basal expression levels in Control were 1 ± 0.038. By conducting one-way ANOVA, we observed a significant difference among groups [F(3,8) = 354.7, **** *p* < 0.0001] (Figure 4E). LPS administration upregulated the expression levels of TNFα (57.36 ± 17.62, **** *p* < 0.0001, compared to Control) (Figure 4E). Nevertheless, AM11920 did not affect the LPS-induced elevated levels of TNFα, neither at the smaller dose of 0.5 μΜ (51.92 ± 1.713, *p* > 0.05, compared to LPS; **** *p* < 0.0001, compared to Control), nor at the highest dose of 1 μM (48.26 ± 3.471, *p* > 0.05, compared to LPS; **** *p* < 0.0001, compared to Control) (Figure 4E), suggesting that AM11920 does not affect the pathway implicated on TNFα expression.

Taken together, these results suggest that AM11920 selectively modulates inflammatory signaling pathways in activated microglia. While this compound significantly attenuates LPS-induced IL-6 expression at higher concentrations, it does not affect TNFα production, indicating that its anti-inflammatory actions may preferentially target specific downstream signaling mechanisms rather than globally suppressing pro-inflammatory cytokine expression.

### 3.5. AM11920 Protects Primary Neurons Against AMPA-Induced Excitotoxicity In Vitro

Primary neuronal cultures were employed to confirm the toxic effect of AMPA and the protective effect of AM11920 that we observed in vivo. NeuN was used to stain neuronal nuclei, MAP2 to stain the dendritic arborization of neurons, and cl. casp.3 to verify the presence of apoptotic cell death in our cultures.

**Figure 5:**
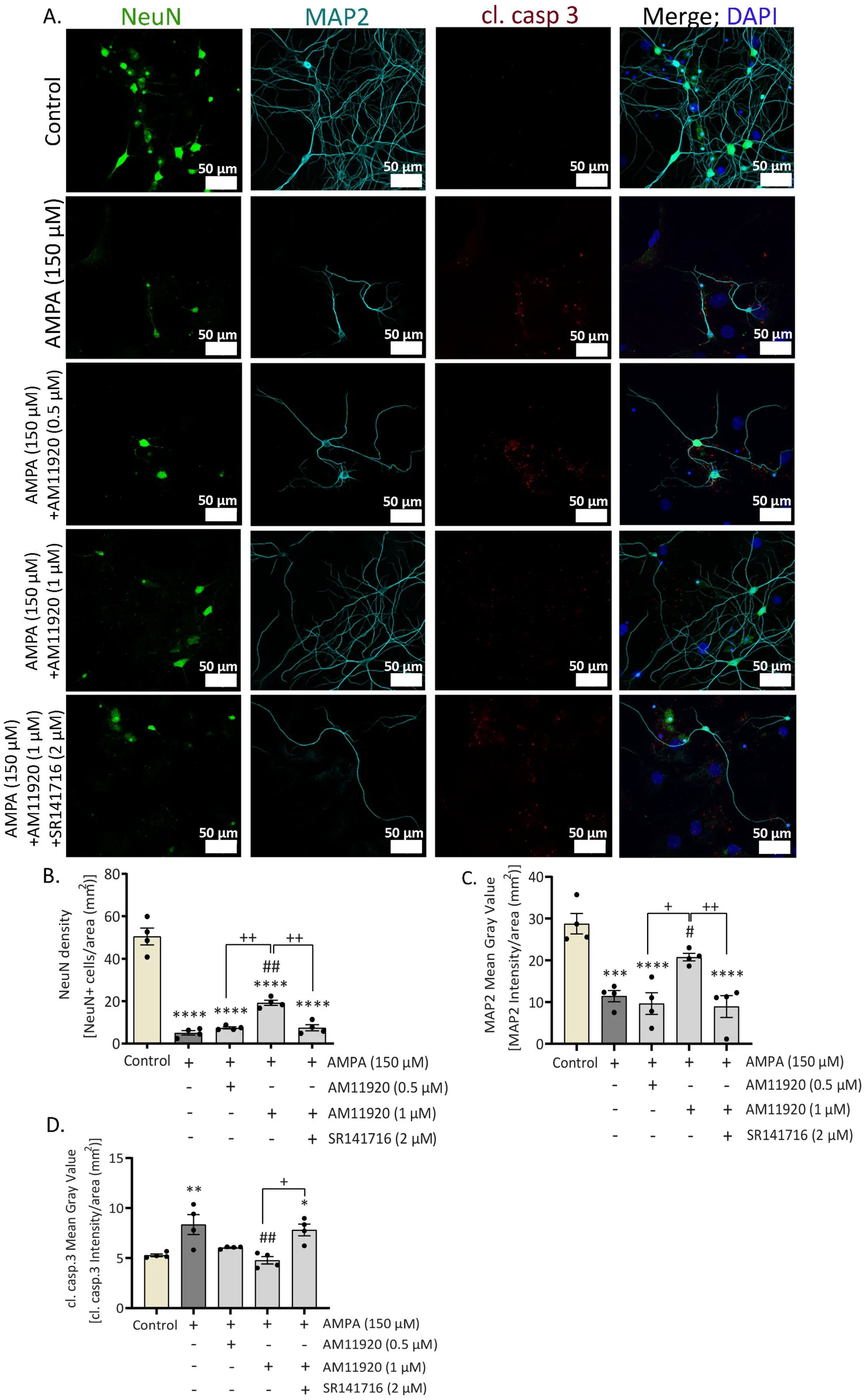
AM11920 protects primary neurons against AMPA-induced excitotoxicity in vitro. (**A**) Representative photomicrographs of Control, AMPA (150 μΜ), AMPA + AM11920 (0.5 and 1 μΜ), and AMPA + AM11920 (1 μΜ) + SR141716 (2 μΜ) stained for NeuN (green), MAP2 (cyan), cl. casp. 3 (red) and DAPI (blue). Scale bar: 50 μm. (**B**,**C**) Quantification analysis of NeuN density (**B**) and MAP2 immunoreactivity (**C**) in Control (*n* = 4), AMPA (*n* = 4), AMPA + AM11920 (0.5 and 1 μΜ) (*n* = 4), and AMPA + AM11920 (1 μΜ) + SR141716 (2 μΜ) (*n* = 4). (**D**) Quantification analysis of cl. casp. 3 immunoreactivity in the same groups (*n* = 4 for all groups). Data are expressed as mean ± SEM. One-way ANOVA was used for statistical comparison, followed by Tukey’s post hoc analysis for multiple comparisons. * *p* < 0.05, ** *p* < 0.01, *** *p* < 0.001, **** *p* < 0.0001 compared to Control; # *p* < 0.05, ## *p* < 0.01, compared to AMPA; + *p* < 0.05, ++ *p* < 0.01, compared to AM11920 treatment.

Multiple comparisons analysis showcases that AMPA administration to neurons leads to a significant decrease in the NeuN density (5.040 ± 2.067, **** *p* < 0.0001), compared to Control group (50.47 ± 7.957) (Figure 5A,B). One-way ANOVA for NeuN density revealed a statistically significant difference among the groups [F(4,15) = 87.79, **** *p* < 0.0001] (Figure 5B). Co-administration of AM11920 with AMPA at the dose of 0.5 μΜ did not suppress the AMPA-induced loss of NeuN (7.385 ± 0.991, *p* > 0.05, compared to AMPA; **** *p* < 0.0001, compared to Control) (Figure 5A,B), but manage to partially block the loss of NeuN at the highest dose (19.24 ± 2.385, ## *p* = 0.0014, compared to AMPA; **** *p* < 0.0001, compared to Control) (Figure 5A,B). Co-treatment of neurons with AM11290 at the highest effective dose (1 μM) and the CB1R antagonist, SR141716, suppressed the protective properties of AM11920 on NeuN (7.447 ± 2.864, *p* > 0.05, compared to AMPA; **** *p* < 0.0001, compared to Control; ++ *p* = 0.0071, compared to AM11920 1 μΜ) (Figure 5A,B).

Regarding MAP2 mean gray value, one-way ANOVA displayed a statistically significant difference among groups [F(4,15) = 16.71, *p* < 0.0001] (Figure 5C). AMPA treatment significantly reduced MAP2 positive dendrites, as seen by the reduced mean gray value (11.44 ± 2.710, *** *p* = 0.0003, compared to Control), compared to Control (28.77 ± 4.902) (Figure 5A,C). Treatment with AM11920 at the highest concentration of 1 μM was able to block the effect of AMPA in MAP2 mean gray value (20.78 ± 1.804, # *p* = 0.0457, compared to AMPA; *p* > 0.05, compared to Control) (Figure 5A,C), while the lower concentration of 0.5 μΜ did not have such a protective effect on MAP2 immunoreactivity (9.656 ± 5.197, *p* > 0.05 compared to AMPA; *p* < 0.0001, compared to Control) (Figure 5A,C). Pre-treatment of neurons with the CB1R antagonist, SR141716, abolished the protective effect of AM11920 1 μΜ on MAP2 (8.936 ± 5.212, *p* > 0.05, compared to AMPA; **** *p* < 0.0001, compared to Control; ++ *p* = 0.0091, compared to AM11920 1 μΜ) (Figure 5A,C).

Analysis using one-way ANOVA identified statistically significant group differences in cl. casp. 3 immunoreactivity, as well [F(4,15) = 8.172, ** *p* = 0.0010] (Figure 5D). Post hoc analysis demonstrated an increase in cl. casp. 3 mean gray value, induced by AMPA (8.348 ± 1.998, ** *p* = 0.0092, compared to Control), compared to Control neurons (5.270 ± 0.275) (Figure 5A,D). AM11920 at the concentration of 1 μM managed to fully block the AMPA-induced upregulation in cl.casp. 3 mean gray value (4.774 ± 0.733, ## *p* = 0.0027, compared to AMPA; *p* > 0.05, compared to Control), while at the smaller concentration (0.5 μΜ), AM11920 did not suppress cl. casp. 3 immunoreactivity (6.035 ± 0.124, *p* > 0.05, compared to AMPA; *p* > 0.05 compared to Control) (Figure 5A,D). Again, co-injection of AM11920 with SR141716, diminished the AM11920-iduced decrease in cl. casp. 3 mean gray value (7.808 ± 1.179, *p* > 0.05, compared to AMPA; * *p* = 0.0352, compared to Control; *p* > 0.05, compared to AM11920 0.5 μΜ; *p* = +0.0103, compared to AM11920 1 μΜ) (Figure 5A,D).

Collectively, these findings demonstrate that AMPA induces severe excitotoxic injury in primary neuronal cultures, induced severe neuronal loss, as evidenced by decreased NeuN and MAP2 immunoreactivity and upregulated cl. casp. 3. Treatment with the inhibitor, AM11920, upregulated the endogenous 2-AG levels, which acting through CB1R, protected neurons by reducing cell death.

### 3.6. AM11920 Protects Primary OPCs Against AMPA-Induced Excitotoxicity In Vitro

OPCs are known to be highly vulnerable to AMPA-mediated toxicity [41,44]. For this reason and given the fact that in vivo we only test the effect of AMPA on mature oligodendrocytes, we decided to examine the effect of AMPA and AM11920 on primary OPCs by measuring the density of PDGFRα+ cells and the density of double cl. casp.3+ and PDGFRα+ OPCs.

**Figure 6:**
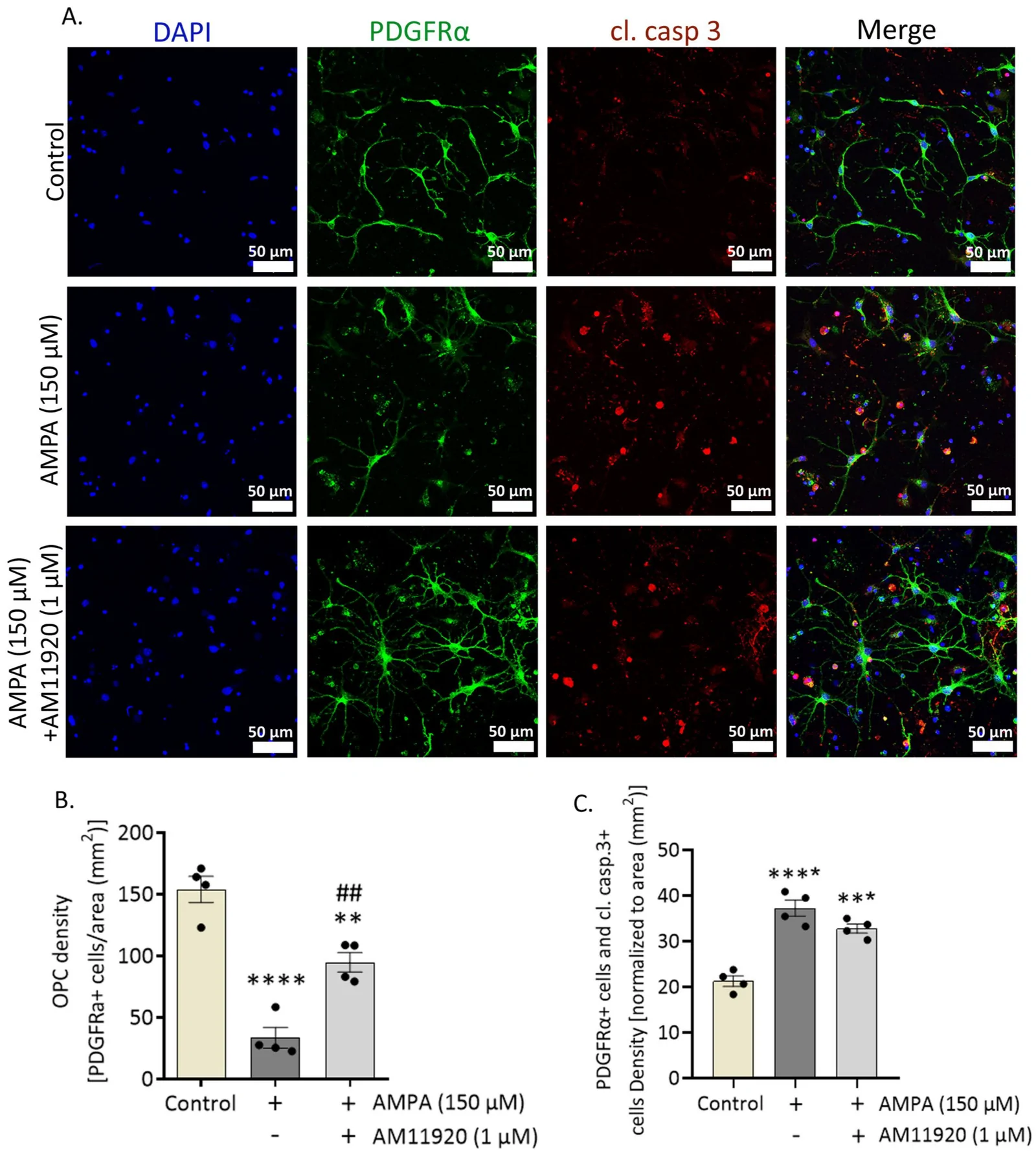
AM11920 protects primary OPCs against AMPA-induced excitotoxicity in vitro. (**A**) Representative photomicrographs of Control, AMPA (150 μΜ), and AMPA + AM11920 (1 μΜ)-treated OPCs, stained for PDGFRα (green), cl. casp. 3 (red) and DAPI (blue). Scale bar: 50 μm. (**B**,**C**) Quantification analysis of PDGFRα density (**B**) and cl. casp. 3 density (**C**) in Control (*n* = 4), AMPA (*n* = 4), and AMPA + AM11920 (*n* = 4)-treated OPCs. Data are expressed as mean ± SEM. One-way ANOVA was used for statistical comparison, followed by Tukey’s post hoc analysis for multiple comparisons. ** *p* < 0.01, *** *p* < 0.001, **** *p* < 0.0001 compared to Control; ## *p* < 0.01, compared to AMPA.

Density of PDGFRα cells in control culture was 153.9 ± 21.34. AMPA administration induced a massive decrease in PDGFRα density (33.50 ± 16.78, **** *p* < 0.0001, compared to Control) (Figure 6A,B), indicating that these cells are sensitive to AMPA excitotoxicity. One-way ANOVA revealed significant differences [F(2,9) = 43.89, **** *p* < 0.0001] (Figure 6B). AM11920 at a concentration of 1 μΜ manage to partially block the AMPA-induced loss of PDGFRα+ OPCs (94.85 ± 15.93, ## *p* = 0.0026, compared to AMPA; ** *p* = 0.0033, compared to Control) (Figure 6A,B), suggesting a cytoprotective effect.

Regarding cl. casp. 3 density, there was also a significant effect of treatment [F(2,9) = 37.28, **** *p* < 0.0001] (Figure 6C). The number of cl. casp. 3+ OPCs in control cells was 21.25 ± 2.324. AMPA induced an upregulation in the number of cl. casp. 3+ OPCs (37.25 ± 3.532, **** *p* < 0.0001, compared to Control), indicating that the loss of OPCs is in part due to the activation of apoptotic signaling. AM11920 administration, which partially blocked the AMPA-induced loss of PDGFRα+ OPCs, did not attenuate the AMPA-induced upregulation of cl. casp. 3 in OPCs (32.78 ± 2.015, *p* > 0.05, compared to AMPA; *** *p* = 0.0005, compared to Control) (Figure 6A,C). These in vitro experiments indicate that AM11920 protects primary OPCs against AMPA-induced excitotoxicity through a non-apoptotic-dependent mechanism.

## 4. Discussion

The data presented in the current study provide evidence supporting a neuroprotective role of enhanced ECS signaling. Briefly, it is shown that amplification of endogenous 2-AG signaling by the dual MAGL/ABHD6 inhibitor AM11920 partially blocks the loss of neuronal cells in an in vivo model of AMPA-induced excitotoxicity. This finding is also corroborated by in vitro evidence, displaying that AM11920 rescues primary neurons from AMPA-induced death and abolishes cl. casp. 3 immunoreactivity, via a CB1R-dependent mechanism. These neuroprotective properties were accompanied by a decrease in microglial density and GFAP immunoreactivity in vivo at the lesion site, while in vitro AM11920 reduced microglial reactivity and the expression of the pro-inflammatory cytokine IL-6, pointing to an attenuation of inflammation. In addition, AM11920 induced an increase in the number of mature oligodendrocytes at the lesion site in vivo, while it protected OPCs from the AMPA insult in vitro.

Consistent with these findings, AMPA injection into the cortex led to increased microgliosis, (Figure 1A–C), while microglial cells underwent an intense morphological transition with thickening of their cell body, suggesting a shift to their activation state, as well. This is in accordance with studies demonstrating that in response to excitotoxic stimuli, microglial cells increase their proliferation rate at the lesion site [10,11,46], while also displaying thickening of their soma [11]. Reactive microglial cells act by either releasing pro-inflammatory mediators such as TNFα [14,47] or by directly releasing glutamate [48,49], which potentiates excitotoxic damage to neurons.

Chronic administration of AM11920 reduced microglial accumulation at the lesion (Figure 1A–C). The remaining microglial cells at the site seemed to adopt a control-like morphology (Figure 1B), a finding that can be considered as a reduction in their reactivity. Rogove and Tsirka [13], demonstrated that the attenuation of excitotoxin-induced microglial activation is crucial for the reduction in kainate-induced neuronal degeneration in the mouse hippocampus. Interestingly, the inflammatory actions of excitotoxin-induced activated microglia do not require a chronic basis to affect neurons, since even in acute conditions they can be detrimental for neuronal survival [50].

The reduction in microglial density reported here is in conjunction with previous studies showing that AM11920 raised endogenous 2-AG levels and reduced microglial reactivity under acute [32] and chronic conditions [33] of AMPA-induced excitotoxicity in rat retina. Similarly to this, exogenous administration of 2-AG was shown to protect axons in a mouse model of experimental autoimmune encephalomyelitis (EAE), by inducing ramification of activated microglia and increasing the percentage of Arginase1+ (Arg1+) microglial cells, which are characterized as alternatively activated cells and possess anti-inflammatory and neurorepair properties [51]. In line with these findings, in a mouse model of Theiler’s murine encephalomyelitis virus-induced demyelination disease (TMEV-IDD), i.p. administration of either 2-AG or UCM-03025, a reversible inhibitor of 2-AG hydrolysis, reduced the accumulation, reactivity, and morphological complexity of microglial cells, while cells also polarized towards an anti-inflammatory phenotype by reducing the expression of several pro-inflammatory cytokines and increasing the expression of anti-inflammatory markers [52]. Other studies have indicated that blockade of MAGL [31] and ABHD6 [30] attenuated microglial reactivity and ameliorated disease severity in animal models of demyelination. Accumulating evidence indicates that the suppression of microglial-mediated inflammation is mostly CB2R-mediated [33,52]. The crucial role of CB2R in regulating microglial cell responses is also supported by the wide expression of CB2R on microglia [24] and the upregulation in its expression upon microglial activation [53,54].

The in vivo findings regarding the effects of AM11920 on microglial activation are further supported by complementary in vitro data. Classical activation of mouse primary microglial cells with LPS, led to an increase in the immunoreactivity of CD68, without affecting the total density of the cells (Figure 4A–C). CD68 is a marker of microglia phagocytic activity and therefore reactivity, which reflects lysosomal activity and is commonly upregulated in reactive microglia [45,55]. In parallel, LPS also induced an upregulation in the expression of the pro-inflammatory cytokines IL-6 and TNFα (Figure 4D,E), indicating a shift towards a pro-inflammatory state. This finding is consistent with reports in the literature, indicating that LPS, through the engagement of nuclear factor κB (NFκB) signaling, upregulates the mRNA and protein levels of certain pro-inflammatory mediators [56,57]. The enhanced 2-AG signaling in microglial cultures, induced by treatment with AM11920 inhibitor, reduced microglial reactivity, as shown by the reduced CD68 immunoreactivity (Figure 4A,C) and partially suppressed the LPS-induced elevated expression of IL-6 (Figure 4D), indicating a suppression of the inflammatory response. The reduced immunoreactivity of CD68, observed in the AM11920-treated microglial cells, reflects a less phagocytically reactive phenotype. Similarly to this, it has been shown that genetic MAGL knockdown in primary microglia reduces the Fcγ receptor-mediated phagocytosis, while MAGL expression increases this activity in BV-2 microglial cell line [58]. In line with our in vivo results, in vitro evidence suggests that application of 2-AG promotes a shift in classically activated microglial cells towards an alternatively activated phenotype, with upregulated expression of anti-inflammatory markers like Arg1 and SOCS3 involving the activation of both CB1R and CB2R [59]. These data are also confirmed for AEA, with studies showing that it can reduce the inflammatory activation of microglia and stimulate their inflammatory capabilities [59,60,61]. Despite the reduction in IL-6 expression, AM11920 did not suppress the elevated expression of TNFα, 4 h after LPS treatment (Figure 4E). This effect suggests that inhibition of 2-AG metabolism at this time point might produce targeted immunomodulatory effects in microglia rather than broad suppression of LPS-induced inflammatory signaling. In support of this hypothesis, differential regulation of IL-6 and TNFα downstream of TLR4 has been previously described [57] and may explain the cytokine-specific effect observed in our case. Although both IL6 and TNFα can be induced through the canonical TLR4 pathway, it is possible that their expression can be differentially influenced by the contribution of NfκB-dependent pathways and Mitogen-activated protein kinase (MAPK) signaling, as well as post-translational mechanisms regulating cytokine mRNA stability. Therefore, AM11920 may preferentially modulate signaling processes that influence IL6 expression, without inducing an analogous effect on TNFα expression under the experimental conditions examined here [62] (Supplementary Figure S3).

Regarding astrocytes, AMPA administration induced an upregulation in GFAP immunoreactivity in the cortical region surrounding the injection site (Figure 1A,B,D). In conjunction with our data, substantial evidence indicates that excitotoxic insults such as glutamate, kainic acid and AMPA induce astrocyte reactivity characterized by increased GFAP expression in astrocytes [32,63,64,65]. In pathological conditions, such as excitotoxicity, reactive astrocytes adopt a pro-inflammatory phenotype by releasing cytokines and complement factors, which initially can engage in reciprocal signaling with microglia and eventually potentiate the detrimental impact of neuro-inflammatory injury [18,66,67]. In addition, reactive astrocytes are known to contribute to extracellular glutamate accumulation by downregulating glutamate transporters that are responsible for glutamate re-uptake or by directly increasing the release of glutamate, which amplifies the deleterious effects of excitotoxic signaling and dampens neuronal survival [19,68]. Therefore, reduction in astrocytic reactivity is crucial for limiting the excitotoxic-induced damage. Here, inhibition of 2-AG hydrolysis by chronic administration of AM11920 reduced astrocytic activation (Figure 1A,B,D). This is in line with evidence indicating that genetic or pharmacological inhibition of MAGL reduces astrocytic reactivity and, therefore, signaling pathways related to inflammatory activity and confers protection to neurons in experimental models of neurodegenerative disease [32,69,70,71].

Beyond cannabinoid signaling-dependent control of inflammatory activity, inhibition of MAGL activity also reduces inflammation through modulation of arachidonic acid metabolism. Arachidonic acid resulting from 2-AG hydrolysis by MAGL constitutes a substrate for the formation of pro-inflammatory prostaglandins and other inflammatory eicosanoids [72,73]. Inhibition of MAGL activity could therefore have an anti-inflammatory potential by reducing arachidonic acid and attenuating the production of the pro-inflammatory prostaglandins. Indeed, it has been demonstrated that the anti-inflammatory and neuroprotective properties resulting from MAGL inhibition, are attributed to the decrease in neuro-inflammatory prostaglandin levels [69,74,75,76]. Therefore, the reduction in inflammatory activity exerted in our case by AM11920 might be a result of not only cannabinoid signaling but also of a concomitant reduction in prostaglandin synthesis.

AMPA-mediated excitotoxicity led to extensive neuronal loss both in vivo in mouse cortex (Figure 2A,C) and in vitro in primary neuronal cultures, as seen by the reduced NeuN density (Figure 5A,B) and loss of MAP2+ immunofluorescence (Figure 5A,C). These results are in agreement with studies suggesting that excessive AMPA induces neuronal death in animal models of disease [77,78,79,80]. Apoptotic cell death is one of the major types of cell death that neurons undergo in AMPA-mediated excitotoxicity due to the activation of calcium-dependent caspases, including cl. casp. 3 [77,81]. Interestingly, blockade of MAGL and ABHD6-mediated hydrolysis of 2-AG by AM11920 attenuated the neurotoxic effects of AMPA in vivo by limiting the area of neuronal damage (Figure 2A–C). Similarly, in vitro, AM11920 provided partial protection to cortical neurons against AMPA (Figure 5A,B), by attenuating cl. casp. 3 immunoreactivity (Figure 5A,D), while it also maintained their dendritic complexity (Figure 5A,C). Interestingly, these effects were CB1R-mediated, as pre-treatment of primary neurons with the CB1R antagonist, SR141716, abolished the observed neuroprotective properties of AM11920 (Figure 5). The only other known neuroprotective properties of AM11920 come from the report of Kokona et al. [32], stating that AM11920 protected nitric oxide synthase (NOS)-expressing amacrine cells subjected to AMPA excitotoxicity in rat retina. Data about the neuroprotective properties of AM11920 reinforce previous evidence, indicating that 2-AG reduces the number of degenerating neurons in a model of NMDA-induced excitotoxicity in organotypic hippocampal slice cultures [82]. Similarly, the beneficial actions of the MAGL inhibitor, JZL184, in restoring hippocampal structural and synaptic integrity and improving spatial learning and memory, have been established in the 5XFAD genetic model of AD [69]. Additionally, JZL184 was shown to restore functional recovery of abnormal synaptic and intrinsic neuronal properties [83] and improve neurobehavioral impairment [84] in models of TBI.

Unexpectedly, despite extensive neuronal damage, as evidenced by the absence of NeuN+ nuclei at the site of AMPA injection (Figure 2A), MBP immunoreactivity remained detectable and appeared unaffected by the excitotoxic insult or AM11920 within the necrotic core of the lesion (Figure 2A,B,D). This finding contradicts data in the literature supporting the finding that excitotoxic insults, such as NMDA, lead to extensive myelin damage in the external capsule of mice seven days post-lesion [85]. Nevertheless, the unaffected MBP immunoreactivity should not be interpreted as preserved myelin integrity, since that would require extensive ultrastructural analysis of myelin by scanning electron microscopy. In further support of this, upon CNS injury, MBP undergoes several post-translational modifications, such as citrullination, which alter its structure and affect myelin compaction [86], and these functional and structural changes are not evident in immunostaining. Persistent MBP immunoreactivity, shown in the present manuscript, might represent residual myelin debris due to proteolysis [87], which also coincides with the absence of microglia and astrocytes at the necrotic core of the AMPA-treated animals (Figure 1A), suggesting a potentially defective clearance of myelin residues after damage [88,89,90]. Our data cannot distinguish if small increases in myelin occur, as MBP is a highly abundant protein. In addition, as CC1 is expressed before mature oligodendrocytes produce myelin, it is possible that in our case, an increase in a transitional population that is mature but not yet myelinating occurs, especially since the tissue was obtained soon after treatment. Studies have demonstrated temporally distinct changes in oligodendrocyte population and myelin-associated markers, supporting the possibility that cellular responses and myelin remodeling do not occur synchronously [91].

Long-term enhancement of endocannabinoid signaling using inhibitors of endocannabinoid metabolism leads to the development of tolerance. This effect depends on the duration of the treatment and dose of the drug [43,44,92]. The reduction in signaling capability of CB1R diminishes the therapeutic efficacy of the cannabinoid used, a property that reevaluates the use of cannabinoids in chronic disorders. To mitigate this risk, a relatively low dose of AM11920 (0.5 mg/kg) was employed in the present study which did not alter CB1R protein expression in the lesion area after seven days of treatment (Figure 2E,F). This finding agrees with previous reports about the effects of AM11920 in a retinal model of excitotoxicity [33] as well as [43] reporting that chronic use of JZL184, a MAGL inhibitor, did not induce tolerance when administered in doses ≤8 mg/kg.

Regarding mature oligodendrocytes, AMPA did not affect the density of CC1+ cells at the lesion site in vivo (Figure 3) but increased the number of PDGFRα+ cells (Supplementary Figure S2). At first sight, this seems like a rather contradictory finding as numerous in vitro and in vivo evidence state that mature oligodendrocytes are highly vulnerable to AMPA-mediated excitotoxicity [42,93,94,95]. Timing is a crucial variable that might explain the absence of difference in CC1 density between control and AMPA-treated animals. More specifically, in pathological conditions, such as demyelinating disorders, the initial loss of oligodendrocytes is followed by rapid proliferation, as indicated in our case too, and migration of OPCs towards the affected area to repair the damage [96]. Another explanation might be attributed to the intrinsic resilience of oligodendrocytes to cell death. It has been shown that human-derived oligodendrocytes, subjected to glutamate excitotoxicity in vitro, retracted their processes and underwent transcriptional changes, indicative of injury, but remained viable six days after the addition of glutamate [97]. Bame et al. [98] in a recent study provided further support for oligodendrocyte resilience. The authors employed optically targeted DNA damage that selectively caused single-cell demyelination, which leads to loss of mitochondria in mature oligodendrocytes, but cells remained alive for months before cell death was evident [98]. The previously reported findings about the vulnerability of oligodendrocytes in AMPA excitotoxicity [42] were verified by our in vitro data (Figure 6A,B). We observed a reduction in OPC cell density which was reversed by AM11920 without decreasing apoptotic death, thus potentially involving non-apoptotic mechanisms. These could include necrosis, necroptosis, and autophagy-dependent cell death among others, often triggered by inflammation, oxidative stress or metabolic dysfunction [99].

Even though the density of CC1+ cells was not affected by AMPA injection, administration of AM11920 induced a significant increase in the density of the cells at the lesion site, an effect which in combination with the reduced population of PDGFRα+ OPCs reflects an enhancement in the differentiation, and/or recruitment of oligodendrocytes. This effect is in agreement with the existence of a fully functional ECS in oligodendroglia, which is involved in the regulation of several physiological responses of oligodendrocytes [100,101]. 2-AG, in particular, is implicated in all of these processes. It has been proposed that a basal 2-AG tone is necessary for the proliferation of OPCs, while addition of the MAGL inhibitor JZL184 increased OPC proliferation via CB1 and CB2 receptors and the activation of the Protein Kinase B/mechanistic Target of Rapamycin (Akt/mTOR) pathway [102]. Similarly, enhancement of 2-AG signaling enhances migration of OPCs by potentiating their chemotaxis and chemokinesis [103]. Facilitation of 2-AG signaling by inhibitors of its hydrolysis has also been proven to enhance the differentiation of OPCs into mature oligodendrocytes [104,105,106,107], by activating the extracellular signal-regulated kinase (ERK)/MAPK pathway in a CB1R/CB2R-dependent mechanism [104], while disruption of 2-AG synthesis by inhibiting its biosynthetic enzymes has the opposite effect in the maturation process [104]. In the present study, administration of AM11920, reduced the AMPA-induced loss of OPCs in vitro (Figure 6A,B), which, to our knowledge, is the first report about the effect of AMPA and cannabinoid treatment in OPCs in vitro. In agreement with our data, Bernal-Chico et al. [31] reported that MAGL blockade, by JZL184, protected differentiated oligodendrocytes in culture subjected to AMPA excitotoxicity by attenuating Ca^2+^ influx, mitochondrial dysfunction, and accumulation of reactive oxygen species, while these effects were abolished by the CB1R antagonist, AM281. Analogous effects about the cytoprotective properties mediated by the ECS in OPCs, come from the study of Molina-Holgado et al. [108], where it was shown that CB1R agonists activated the phosphoinositide 3-kinase (PI3K)/Akt pathway, reduced caspase activation and rescued OPCs in vitro, subjected to serum deprivation. Our in vitro observation that the protection by 2-AG involves a caspase-independent mechanism fits well with evidence indicating that oligodendrocytes subjected to AMPA excitotoxicity undergo both caspase-dependent and independent death [109]. Interestingly, administration of caspase inhibitors is not sufficient to block AMPA-induced cell death [109], suggesting that apoptotic death, despite its presence, is not primarily responsible for oligodendrocyte loss, which is also supported by other studies showing that upon AMPA excitotoxicity, oligodendrocytes die predominantly by necrosis [94].

Several limitations of the present study should be acknowledged. Firstly, although our findings support the involvement of CB1R in the neuroprotective actions of AM11920, the contribution of CB2R was not evaluated by the employment of a selective CB2R antagonist. Thus, the extent to which CB2R mediated the neuroprotective and anti-inflammatory actions of AM11920 remains to be established. Nevertheless, previous reports in a model of AMPA excitotoxicity in rat retina clearly indicate that the anti-inflammatory actions of AM11920 are mostly mediated through the activation of CB2R [32,33]. Secondly, the present treatment paradigm was limited to seven consecutive days and therefore potential changes in CB1R expression or signaling associated with longer-term administration of AM11920, were not evaluated. Given the chronic nature of excitotoxicity, such studies would be useful to determine whether prolonged inhibition of 2-AG metabolism results in adaptive changes in the ECS that can interfere with the observed protective actions. Another limitation of the present study is the dose of AMPA employed for the in vitro experiments (150 μΜ), which represents a robust excitotoxic challenge and may not directly reflect the concentration of glutamate, observed in vivo under excitotoxic conditions. Therefore, our in vitro findings should be interpreted as cellular responses to excitotoxic stress, rather than as a direct representation of the in vivo exposure. Apart from this, the lack of behavioral assessment represents another limitation of this study and therefore it remains unclear whether the protection observed at the cellular level translates into functional improvement too. Future studies incorporating behavioral assessment of cognitive and sensorimotor function are necessary for establishing the functional relevance of AM11920-mediated protection. Lastly, some experiments were performed in small sample size (*n* = 3 or 4), which may limit statistical power. Nevertheless, the consistency of the observed effects across the in vivo and in vitro experiments provides supportive evidence for the overall conclusions.

## 5. Conclusions

Collectively, the present study demonstrates that pharmacological enhancement of endogenous 2-AG signaling through inhibition of its primary hydrolyzing enzymes, MAGL and ABHD6, by AM11920, confers significant neuroprotection and exerts broad immunomodulatory control over excitotoxic inflammation induced by AMPA. The observed effects likely arise from the combinatory regulation by the ECS of neuronal survival pathways, inflammatory responses mediated by activated glial cells, and oligodendroglia cell function. Importantly, under the conditions examined here, repeated administration of AM11920 did not alter CB1R expression, the principal mediator of 2-AG-dependent neuroprotective signaling. Excitotoxicity is a central pathological feature across a wide spectrum of neurodegenerative and neuro-inflammatory disorders, and effective therapeutic strategies must therefore maintain efficacy under chronic administration. Therefore, finding new pharmaceutical agents that can achieve their therapeutic benefits without inducing tolerance is a step of great importance when choosing an appropriate treatment for excitotoxicity. Further studies are needed to elucidate the exact molecular events that give rise to the neuroprotective and anti-inflammatory actions of AM11920, examine the long-term impact of ECS modulation, and define the impact of AM11920 on oligodendrocyte events, such as proliferation, differentiation, and migration, three key players that govern myelin repair after injury. Despite the existence of some limitations, the present findings underscore the therapeutic promise of targeting endocannabinoid metabolic enzymes as a strategy to modulate inflammation and promote neuronal and oligodendroglia survival in excitotoxic conditions.

## Figures and Tables

**Table 1:** Primers used for qRT-PCR.

GENE	Forward	Reverse
Actin	5′ GCAGCTCCTT- CGTTGCCGGT 3′	5′ TACAGCCCGG- GGAGCATCGT 3′
IL-6	5′ GAGGATACCACTCCCAACAGACC 3′	5′ AAGTGCATCATC- GTTGTTCATACA 3′
TNF-α	5′ GCCTCTTCTCA- TTCCTGCTTG 3′	5′ CTGATGGAG- GGAGGCCATT 3′

## Data Availability

Data used in this manuscript are available by request to the corresponding authors.

## Supplementary Materials

The following supporting information can be downloaded at: https://www.mdpi.com/article/10.3390/biom16101496/s1, Figure S1: Uncropped immunoblots of CB1R expression; Figure S2: AM11920 attenuates the AMPA-induced increase in OPC population at the lesion site in vivo; Figure S3: Putative mechanism by which AM11920 attenuates neuro-inflammation in cortex through increased 2-arachidonoylglycerol (2-AG) signaling.

## Abbreviations

The following abbreviations are used in this manuscript:

2-AG	2-Arachidonoylglycerol
ABHD6	α/β-hydrolase domain-containing 6
AEA	Anandamide
AMPAR	α-Amino-3-hydroxy-5-methyl-4-isoxazolepropionic acid receptor
CB1R	Cannabinoid receptor type 1
CB2R	Cannabinoid receptor type 2
CD68	Cluster of differentiation 68
cl. casp.3	Cleaved caspase 3
CNS	Central nervous system
DMSO	Dimethyl sulfoxide
EAE	Experimental autoimmune encephalomyelitis
ECS	Endocannabinoid system
GFAP	Glial fibrillary acidic protein
Iba1	Ionized calcium-binding adapter molecule 1
IL-1β	Interleukin-1β
IL-6	Interleukin-6
MAGL	Monoacylglycerol lipase
MBP	Myelin basic protein
NeuN	Neuronal nuclei
NMDAR	N-methyl-D-aspartate receptor
PBS	Phosphate-buffered saline
PBST	Phosphate-buffered saline Tween
PDGFRα	Platelet-derived growth factor α
TBI	Traumatic brain injury
TMEV-IDD	Theiler’s murine encephalomyelitis virus-induced demyelination disease
TNFα	Tumor necrosis factor α
