# Supplementary material for: Enhancement of Endocannabinoid Signaling by Dual MAGL/ABHD6 Inhibition Reduces Neurodegeneration and Neuro-Inflammation Following AMPA-Mediated Excitotoxicity
Source: Biomolecules. 2026 Oct 14;16(10):1496. doi: 10.3390/biom16101496 (PMC13702437; doi:10.3390/biom16101496)
Supplement: Supplementary file 1 [file biomolecules-16-01496-s001.zip › biomolecules-4480516-WB Original Image Suppl Figure S1.pdf]

A.

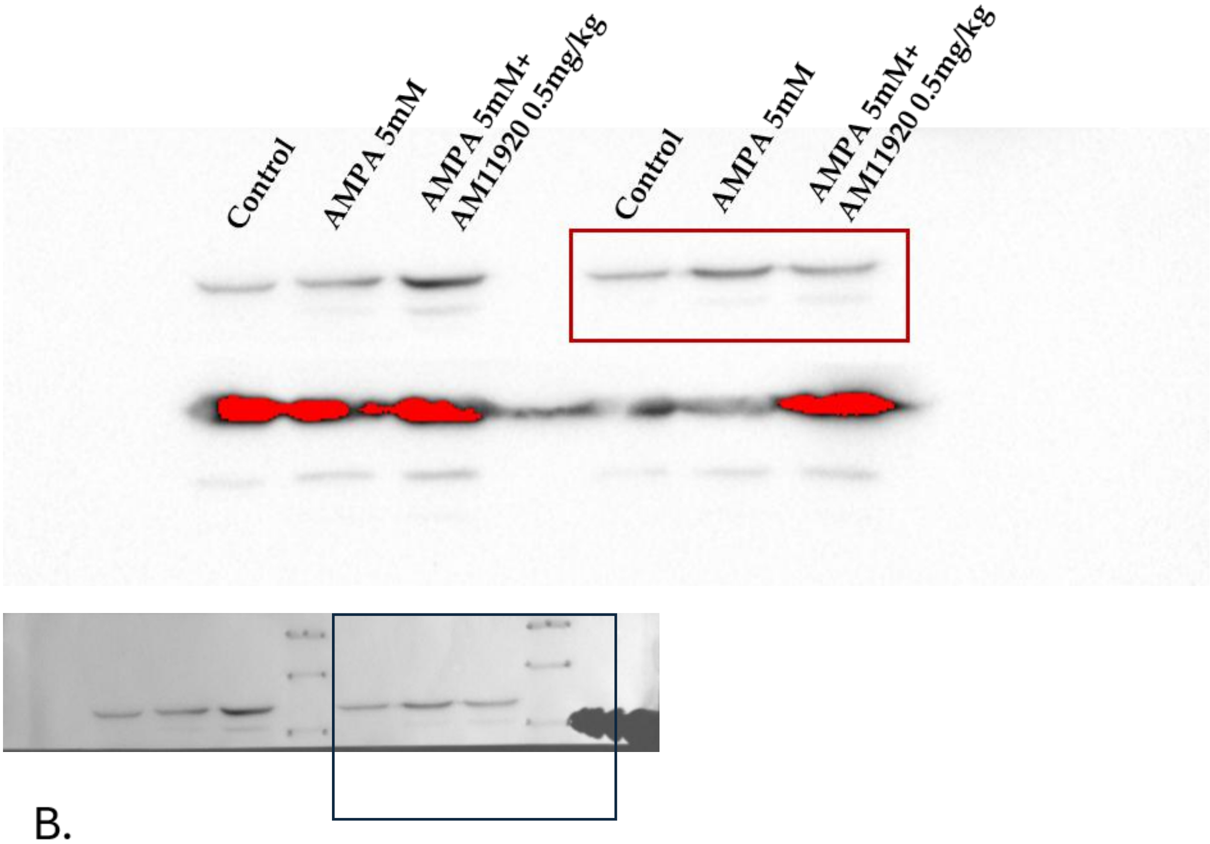

B.

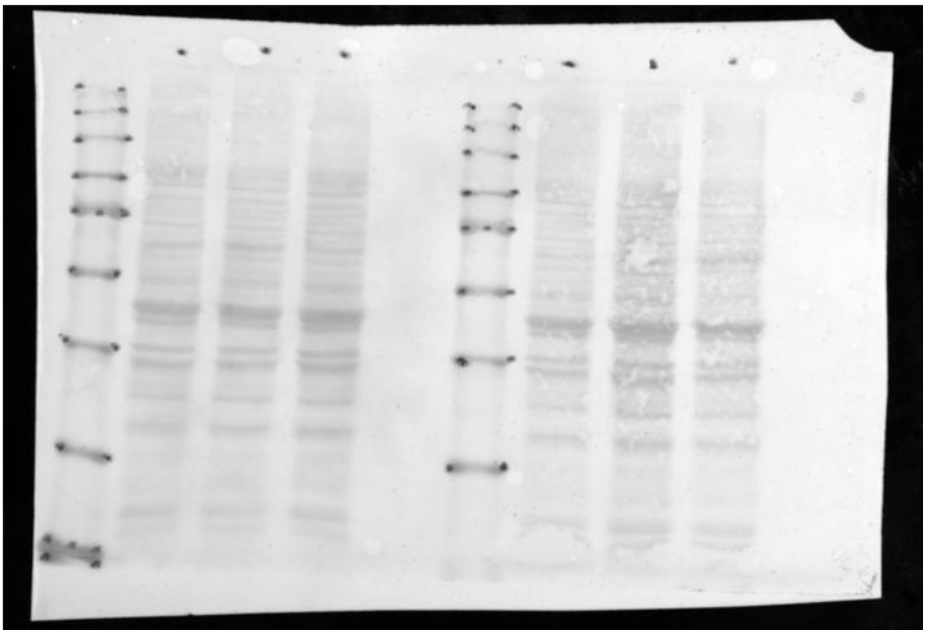

Supplementary Figure S1. A. Uncropped immunoblots of CB1R expression in Control, AMPA (5mM) and AMPA (5mM)+ AM11920 (0.5mg/kg). Bands outlined with the red rectangle represent the ones presented in Figure 2E in the main text. **B.** Ponceau staining of the same membrane.
