# Supplementary material for: Enhancement of Endocannabinoid Signaling by Dual MAGL/ABHD6 Inhibition Reduces Neurodegeneration and Neuro-Inflammation Following AMPA-Mediated Excitotoxicity
Source: Biomolecules. 2026 Oct 14;16(10):1496. doi: 10.3390/biom16101496 (PMC13702437; doi:10.3390/biom16101496)
Supplement: Supplementary file 1 [file biomolecules-16-01496-s001.zip › Suppl files 2 and 3 4480516/Supplementary figure legends 4480516.pdf]

Supplementary figure legends:

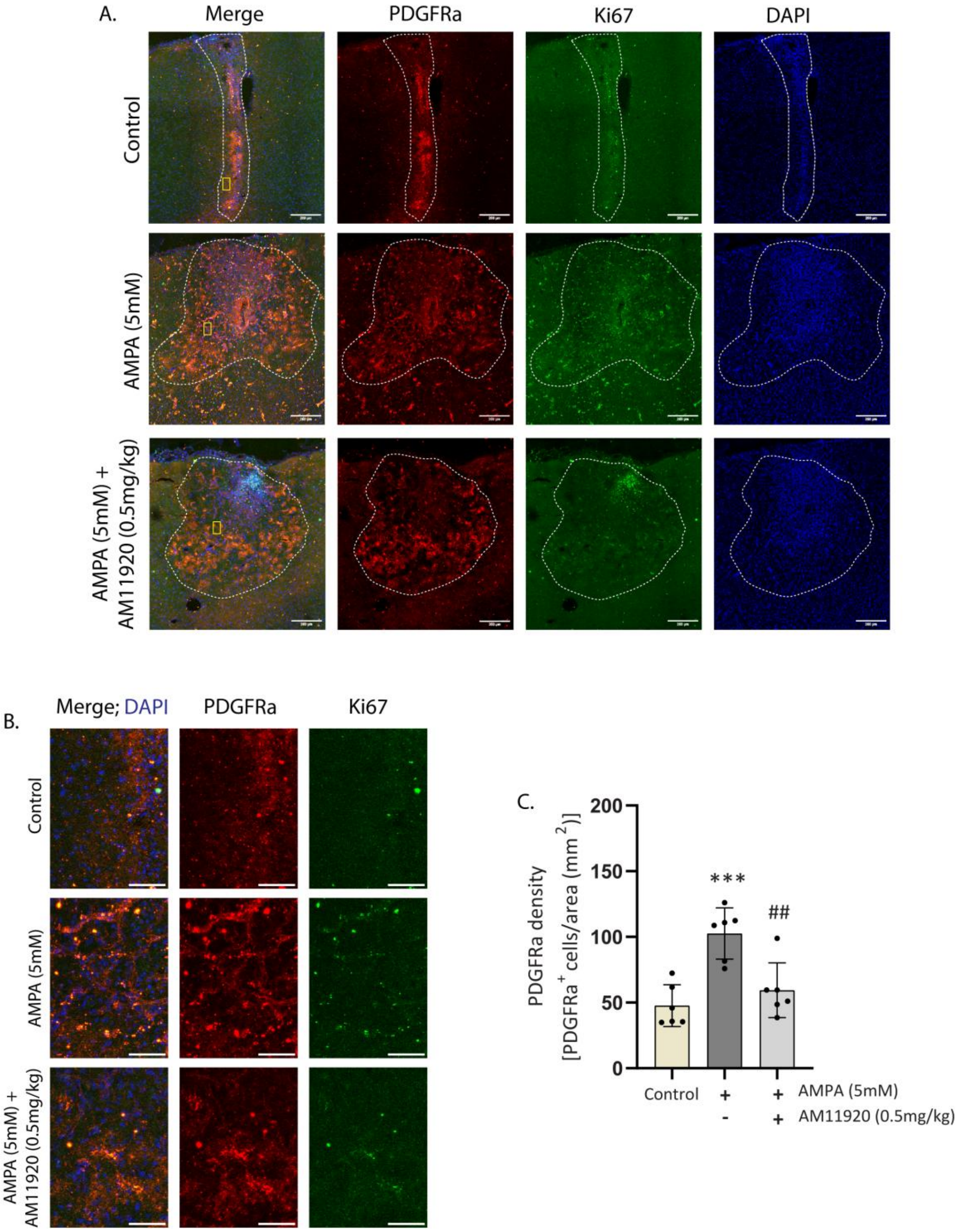

Figure S2 R: AM11920 attenuates the AMPA-induced increase in OPC population at the lesion site *in vivo*.

A. Representative photomicrographs of the M2 region of the prefrontal cortex in Control, AMPA (5 mM), and AMPA+AM11920 (0.5 mg/kg), stained for DAPI (blue), PDGFRa (red) and Ki67 (green). White dashed line marks the lesion site. Scale bar: 200 µm. **B.** Higher magnification of the area outlined in the yellow rectangle. Scale bar: 50 µm. **C.** Quantification analysis of PDGFRa density in Control (n=6), AMPA

(n=6) and AMPA+AM11920 (n=6)-treated animals. Data are expressed as mean ± SEM. One-way ANOVA was used for statistical comparison, followed by Tukey's post hoc analysis for multiple comparisons. ***$p<0.001$, compared to Control; ##$p<0.01$, compared to AMPA.

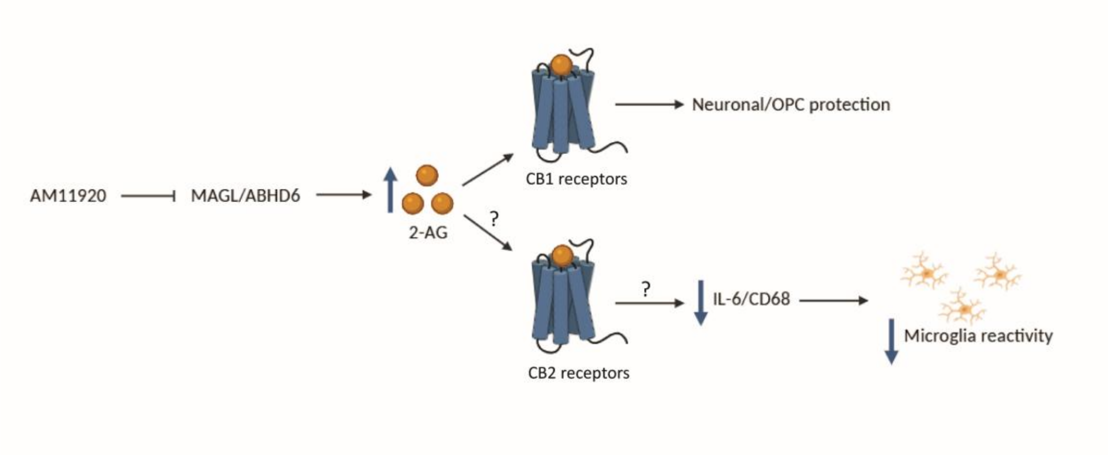

Figure S3 R: Putative mechanism by which AM11920 attenuates neuroinflammation in cortex through increased 2-arachidonoylglycerol (2-AG) signaling.
